# Analysis of the Setomimycin Biosynthetic Gene Cluster from *Streptomyces nojiriensis* JCM3382 and Evaluation of Its α-Glucosidase Inhibitory Activity Using Molecular Docking and Molecular Dynamics Simulations

**DOI:** 10.3390/ijms251910758

**Published:** 2024-10-06

**Authors:** Kyung-A Hyun, Xuhui Liang, Yang Xu, Seung-Young Kim, Kyung-Hwan Boo, Jin-Soo Park, Won-Jae Chi, Chang-Gu Hyun

**Affiliations:** 1 Department of Biotechnology, College of Applied Life Science, Jeju National University, Jeju 63243, Republic of Korea; kah990626@gmail.com (K.-A.H.); khboo@jejunu.ac.kr (K.-H.B.); 2 Jeju Inside Agency and Cosmetic Science Center, Department of Beauty and Cosmetology, Jeju National University, Jeju 63243, Republic of Korea; lxh03036@naver.com (X.L.); iamxuyang1990@gmail.com (Y.X.); 3 Department of Pharmaceutical Engineering and Biotechnology, Sunmoon University, Asan 31460, Republic of Korea; sykim01@sunmoon.ac.kr; 4 Natural Product Informatics Research Center, KIST Gangneung Institute of Natural Products, Gangneung 25451, Republic of Korea; jinsoopark@kist.re.kr; 5 Genetic Resources Assessment Division, National Institute of Biological Resources, Incheon 22689, Republic of Korea

**Keywords:** α-glucosidase inhibitor, biaryl polyketides, molecular docking, molecular dynamics, nonaketide, setomimycin BGC

## Abstract

The formation of atroposelective biaryl compounds in plants and fungi is well understood; however, polyketide aglycone synthesis and dimerization in bacteria remain unclear. Thus, the biosynthetic gene cluster (BGC) responsible for antibacterial setomimycin production from *Streptomyces nojiriensis* JCM3382 was examined in comparison with the BGCs of spectomycin, julichromes, lincolnenins, and huanglongmycin. The setomimycin BGC includes post-polyketide synthase (PKS) assembly/cycling enzymes StmD (C-9 ketoreductase), StmE (aromatase), and StmF (thioesterase) as key components. The heterodimeric TcmI-like cyclases StmH and StmK are proposed to aid in forming the setomimycin monomer. In addition, StmI (P-450) is predicted to catalyze the biaryl coupling of two monomeric setomimycin units, with StmM (ferredoxin) specific to the setomimycin BGC. The roles of StmL and StmN, part of the nuclear transport factor 2 (NTF-2)-like protein family and unique to setomimycin BGCs, could particularly interest biochemists and combinatorial biologists. α-Glucosidase, a key enzyme in type 2 diabetes, hydrolyzes carbohydrates into glucose, thereby elevating blood glucose levels. This study aimed to assess the α-glucosidase inhibitory activity of EtOAc extracts of JCM 3382 and setomimycin. The JCM 3382 EtOAc extract and setomimycin exhibited greater potency than the standard inhibitor, acarbose, with IC_50_ values of 285.14 ± 2.04 μg/mL and 231.26 ± 0.41 μM, respectively. Molecular docking demonstrated two hydrogen bonds with maltase-glucoamylase chain A residues Thr205 and Lys480 (binding energy = −6.8 kcal·mol^−1^), two π–π interactions with Trp406 and Phe450, and one π–cation interaction with Asp542. Residue-energy analysis highlighted Trp406 and Phe450 as key in setomimycin’s binding to maltase-glucoamylase. These findings suggest that setomimycin is a promising candidate for further enzymological research and potential antidiabetic therapy.

## 1. Introduction

**Figure 1:**
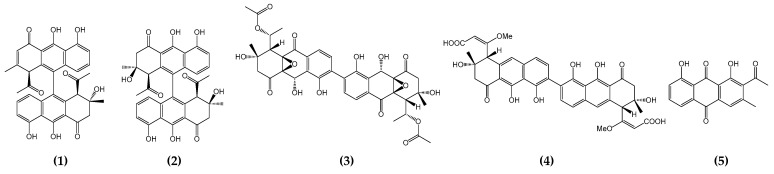
Structures of nonaketide-derived polyketides: setomimycin (**1**), lincolnenins A (**2**), julichromes Q3.3 (**3**), spectomycin A1 (**4**), and huanglongmycin A (**5**).

Aromatic polyketides are primarily produced by actinomycetes and have minimal PKS, comprising two ketosynthases, KSα and KSβ (also known as “chain length factors”), and an acyl carrier protein (ACP). Additional ketoreductases and cyclases/aromatases participate in the formation of the initial polyketide aglycone [1,2]. Over the past 30 years, extensive biosynthetic studies of polyketides have been conducted using actinorhodin, frenolicin, and tetracenomycin C. These studies have culminated in the development of a novel concept, “combinatorial biosynthesis”, which has helped to establish certain “design rules” by identifying the generation of analogs such as DMAC and SEK26 [3,4]. Although aromatic polyketides have been extensively studied for the production of numerous derivatives using combinatorial biosynthesis approaches based on a deep understanding of the biosynthetic pathway, relatively few nonaketide-derived aromatic polyketides have been reported. Among natural nonaketide-derived aromatic polyketides, biaryl compounds are of particular interest owing to their potential for the formation of homo- or heterodimers via oxidative phenol coupling [5,6]. It has been proposed that actinomycete-derived and nonaketide-derived biaryl polyketides are biosynthesized via the Claisen-like condensation of nine malonyl-CoA or one acetyl-CoA and eight malonyl-CoA, as exemplified by setomimycin, spectomycin, julichromes, lincolnenins, and huanglongmycin, among others [7,8]. It is noteworthy that both monomeric and dimeric derivatives have been reported for setomimycin, spectomycin, julichromes, and lincolnenins, whereas huanglongmycin consists only of monomers (Figure 1) [9,10,11,12,13,14]. The pivotal role of P450 enzymes in the formation of the dimeric structure via oxidative phenol coupling of the monomeric structure indicates that the generation of “unnatural” natural biaryl compounds using P450 and ferredoxin may be anticipated [15,16,17].

Meanwhile, nonaketide-derived biaryl polyketides have garnered considerable attention for their efficacy in human health. Setomimycin is produced by *Streptomyces nojiriensis* JCM 3382, *S*. *aurantiacus* JA4570, and *S. justicei* RA-WS2. Their antimicrobial and anticancer activities have also been documented. Recently, industrial *Streptomyces* sp. RA-WS2 was developed with a 16.8-fold improvement in setomimycin production using the Taguchi orthogonal array design [13,14,18,19]. Furthermore, the production of spectomycin B1, a biaryl polyketide, has been documented in *S*. *spectabilis*, a species renowned for its capacity to produce spectinomycin, a broad-spectrum antibiotic that impedes the proliferation of many Gram-positive and Gram-negative bacteria. The structural and small ubiquitin-like modifier (SUMO)ylation inhibition activity of spectomycin B1 and its monomeric forms, spectomycin A1 and A, were studied. This revealed that the dimeric form is important for SUMOylation [12,20]. In contrast, julichromes produced by *S*. *sampsonii* SCSIO 054 are the most frequently reported nonaketide-derived polyketide derivatives. They have attracted attention for their broad spectrum of biological activities, including antimicrobial properties and cytotoxic activity, particularly against various human tumor cell lines [8,11,21]. Huanglongmycin, a nonaketide-derived polyketide that exists only as a monomeric structure, has also been studied in *Streptomyces* sp. CB09001. This has provided useful information for elucidating the overall dimeric structure’s biosynthetic pathway based on the study of biosynthetic enzymes fundamental for monomer production [7,9]. The production of julichrome-type monomers has also been reported in *Streptomyces* sp. 12T061A and 12T061C, which is expected to provide valuable information for elucidating the monomeric biosynthetic pathway [22].

Diabetes mellitus (DM) is a serious endocrine and metabolic disease caused by impaired insulin secretion and insulin resistance. It is characterized by abnormally high blood glucose levels. Long-term elevated blood glucose levels can have deleterious effects on the vascular endothelium, resulting in the development of various complications, including microvascular and macrovascular complications [23]. Alpha-glucosidase catalyzes the conversion of carbohydrates into monosaccharides, providing the energy required for normal human function. In particular, α-glucosidase inhibitors (AGIs) have been demonstrated to not only delay the absorption of carbohydrates but also reduce the peak of postprandial blood glucose [24]. The pharmaceutical use of AGIs has been extensively studied because of their favorable safety profile against hypoglycemia. To date, numerous efforts have developed AGIs, encompassing both natural and synthetic candidates. Nevertheless, only a few substances, including acarbose, voglibose, and miglitol, have demonstrated sufficient efficacy and tolerability and warrant further investigation. Nevertheless, long-term administration of these drugs can cause severe hypoglycemia, weight gain, and gastrointestinal side effects such as abdominal pain, diarrhea, and flatulence [25]. Consequently, the development of novel, safe, and potent AGIs remains a pressing clinical need and highly challenging [24].

In a previous study, we unexpectedly identified a rare bianthracene setomimycin gene cluster during the whole-genome analysis of *S*. *nojiriensis* JCM 3382 and directly confirmed setomimycin production [14]. In this study, we conducted an in silico comparison of the biosynthetic gene clusters of biaryl polyketides centered on setomimycin to expand the scientific knowledge about the rarity of biaryl polyketides and the importance of intermolecular oxidative phenol coupling and their biological activities. Additionally, the α-glucosidase inhibitory activity of setomimycin was evaluated for the first time for biaryl polyketides using molecular docking and molecular dynamics simulation.

## 2. Results

### 2.1. Comparative Analysis of Biosynthetic Gene Clusters between Setomimycin Producing Microorganisms

**Figure 2:**
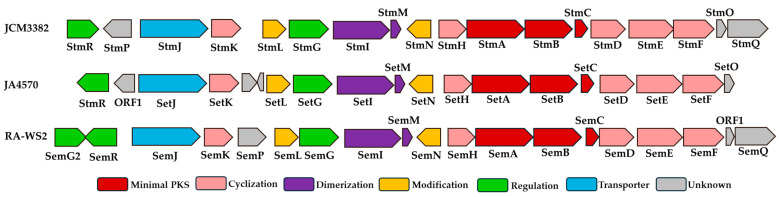
Predicted gene organization of setomimycin BGCs from *S. nojiriensis* JCM 3382 (Stm), *S. aurantiacus* JA4570 (Set), and *S. justiciae* RA-WS2 (Sem). Genes are color-coded according to their proposed functions. Brown, amber, purple, green, blue, and gray represent minimal PKS, cyclization, dimerization, regulation, resistance, and unknown functions, respectively.

As a preliminary step in inferring the setomimycin biosynthetic pathway derived from *S. nojiriensis* JCM 3382, we re-identified 31 BGCs, including those for streptocollin, tambromycin, desferrioxamine B/E, setomimycins, and linearmycins, through genome mining with the latest antiSMASH version 7.1.0 (Table S1) [26]. The existence of setomimycin gene clusters was tentatively inferred through searches conducted at the National Center for Biotechnology Information (NCBI) and antiSMASH analyses. Consequently, we derived the BGCs directly related to setomimycin production from *S. nojiriensis* JCM3382, *S. aurantiacus* JA 4570, and *S. justiciae* RA-WS2 [13,14]. Subsequently, we conducted a comparative analysis of the setomimycin biosynthetic genes of the three actinomycetes to ascertain the boundaries of the gene clusters. As illustrated in Figure 2, JCM3382, JA4570, and RA-WS2 exhibited 14 enzymes (Stm A to StmN) arranged in the same transcriptional orientation and configuration as the setomimycin BGC. Bioactive metabolites, including aromatic polyketides, are produced as machinery encoded by a group of genes, a BGC, which, besides biosynthetic genes, typically includes genes for regulation and self-resistance [27]. Furthermore, setomimycin BGCs contain the StmJ-homologue genes SetJ and SemJ, which are self-resistance genes that function as exporters. The StmP-homologous gene, situated downstream of StmJ, is located upstream of SemJ in the RA-WS2 BGC. This suggests that StmP and SemP are likely to be involved in the biosynthesis of setomimycin. The next step was to ascertain the potential involvement of StmO and StmQ, situated upstream of StmF in the JCM3382 BGC, in setomimycin biosynthesis. As illustrated in Figure 2, a StmO-homologous gene (SetO) is in the JA4570 BGC, and a StmQ-homologous gene (SemQ) is in the RA-WS2 BGC. SetO is translationally coupled to its downstream gene SetF, which lends credence to the hypothesis that it is involved in setomimycin biosynthesis. Furthermore, SemQ is translationally coupled to its downstream genes, ORF1 and ORF1, which are sequentially translationally coupled to SetF. This suggests that SemQ and a potentially unidentified protein, ORF1, are also involved in setomimycin biosynthesis. Set and Sem BGCs also contain inverted StmR, which is a TetR family transcriptional regulator. Consequently, we propose that the boundary points of the *S. nojiriensis*-derived Stm BGC may comprise 18 genes, from StmA to StmR. Nevertheless, the involvement of the putative Stm BGC, including StmO, StmQ, and StmR, in setomimycin biosynthesis must be confirmed by gene disruption or heterologous expression.

### 2.2. Putative Setomimycin Biosynthetic Pathway

#### 2.2.1. Minimal PKS

The initial stage of aromatic polyketide biosynthesis involves loading of the starter unit, which is predominantly acetate, onto ACP. Even setomimycin, produced by *S. nojiriensis* JCM3382, initiates the condensation of one molecule of acetyl-CoA and eight molecules of malonyl-CoA to form a monomeric polyketide intermediate. Type II PKSs have been used up to now to naturally synthesize aromatic polyketides with chain lengths ranging from C-10 to C-30. In recent years, new aromatic polyketides with different carbon chain lengths have been investigated [2]. The biaryl polyketides setomimycin, lincolnenins, julichromes, spectomycin, and huanglongmycin exemplify this trend. Minimal PKS refers to the minimal set of enzymes required for the basic assembly of the polyketide backbone. The minimal PKS, which is responsible for the synthesis of the polyketide core, collectively known as the minimal cassette, has the potential for application in synthetic biology. The minimal cassette provides polyketides of varying chain lengths that interact with other enzymes responsible for different cyclization patterns [2,28]. A typical type II PKS contains a ketosynthase (KS) enzyme that forms a heterodimer with its chain length factor (CLF) partner. The KS-CLF heterodimer regulates the number of condensation reactions that occur during the extension of the polyketide chain, which is exclusively catalyzed by the ACP. The resulting poly-β-keto intermediate is subsequently converted to the backbone of an aromatic polyketide via cyclization, which is catalyzed by a modifying enzyme [1,2,3,4,5,28]. Therefore, setomimycin is also a representative example of a typical type II PKS derivative, comprising β-ketoacyl synthase α (KSα) StmA, β-ketoacyl synthase β (KSβ) StmB, and ACP StmC. Their assembly is biologically initiated by acetyl-CoA. The initial unit is then expanded by eight malonyl-CoA units to form the C-18 main chain, which is subsequently processed by ketoreductase, aromatase, and cyclase machineries to yield a fundamental polycyclic scaffold. A comparison of the three setomimycin minimal PKSs, two julichromes, and the corresponding enzymes from Lincolnian, spectomycin, and huanglongmycin was performed at the sequence level. This analysis revealed that the nine minimal PKSs exhibited unique identities of up to 94% at the protein level, suggesting that they formed the same polyketidic precursor (Table S2). Moreover, phylogenetic analysis of a cluster of 42 known type II PKS genes whose secondary metabolite products have been structurally characterized indicates that the putative KSβ gene StmB is in the KSβ group for nonaketide biosynthesis (Figure S1).

#### 2.2.2. Ketoreduction and Cyclization

During the monomeric polyketide synthesis of setomimycin, the presence of ketoreductase (KR) is essential for the hydroxyl reduction of the regiospecific carbonyl, which determines the product outcome. As anticipated, the BLASTP search identified StmD, which encodes a unique enzyme belonging to the short-chain dehydrogenase/reductase (SDR) family, in setomimycin BGCs. Furthermore, an HHpred search [29] identified that StmD exhibits high similarity to ActIII (PDB, 2RHC) and HedA (PDB, 3SJU), both of which are well-known KRs with C9 reduction of the 16-carbon poly-β-ketone intermediate and 24-carbon poly-β-ketone intermediate in actinorhodin and hedamycin biosynthesis, respectively. Previous studies indicate that KR may direct the C7–C12 first-ring cyclization and introduce stereochemistry in the polyketide intermediate upon reduction [30,31]. Amino acid sequence comparison indicated that StmD (261 aa) exhibited high identity (64.04%) and similarity (76.03%) with HedA, whereas ActIII exhibited high identity (59.18%) and similarity (75.28%). The characteristic sequence motifs found in SDR enzymes are conserved in StmD, including the cofactor-binding motif TGxxxGxG (StmD residues 12–19) and catalytic tetrad residues D109, S144, Y157, and K161. The NNAG motif, which has been proposed to stabilize the central β-sheet and is highly conserved in other type II polyketide KRs, has also been identified in StmD. The ^93^PGG^95^ motif observed in ActIII is also critically conserved in StmD, leading to S-dominant stereospecificity. The molecular basis of this stereospecificity can be explained by its crystal structure, which suggests that the stereospecificity of StmD may be like that of wild-type ActIII. In contrast, HedA contains a PGG motif substituted with NGG (Figure S2). Sequence alignment results demonstrated that HedA and ActIII possess the SDR fold and exhibit high structural conservation with HedA and ActIII. Nevertheless, the enzymatic activity, interactions with ACP, and stereospecificity of StmD may differ from those of HedA and ActIII, reflecting the differences in substrates, domain architecture, and stereochemical requirements of monomeric setomimycin versus hedamycin and actinorhodin PKSs [32,33]. Stm PKS employs a highly conserved StmD KR that specifically reduces only one C9-carbonyl group of the monomeric setomimycin polyketide chain (Figure 2). Subsequently, the presence of multiple carbonyl groups on the setomimycin polyketide chain provided reactive R-carbon centers that led to intramolecular cyclization.

Regiospecific cyclization of ACP-linked linear poly-β-ketone intermediates represents a pivotal biosynthetic process catalyzed by type II PKSs in conjunction with aromatase/cyclase (ARO/CYC) participation, resulting in the formation of the first ring. Crystal structural and biochemical studies of ARO/CYC have provided compelling evidence that TcmN (tetracenomycins), WhiE (spore pigment), and ZhuI (R1128) can catalyze polyketide cyclization and aromatization, thus advancing our understanding of polyketide cyclization [34]. Nevertheless, numerous type II PKSs contain di-domains of ARO/CYC, which appear to be identical in structure. The di-domain ARO/CYCs were found in both non-reducing and reducing PKSs. In non-reducing systems, such as StfQ of steffimycin, the di-domain ARO/CYCs regiospecifically cyclize a polyketide between C7 and C12, followed by aromatization. In reducing systems (e.g., BexL of BE-7585A), KR-first regiospecifically cyclizes the linear poly-β-ketone from C12 to C7, followed by a highly specific C9-carbonyl reduction. Subsequently, the di-domain ARO/CYC catalyzes the dehydration of the C9 hydroxyl group, followed by first-ring aromatization [35]. The setomimycin BGC also contains StmE, which is, unsurprisingly, an ARO/CYC candidate. The HHpred structure homologue search demonstrated that StmE exhibits high similarity to BexL and StfQ, a well-known di-domain ARO/CYC. Furthermore, StmE exhibited amino acid sequence identities of 56.97% and 39.58% with BexL and StfQ, respectively. Examination of the amino acid sequence of StmE revealed that it was highly conserved with R70 in the monodomains ARO/CYCs TcmN, WhiE, and ZhuI. In contrast, StfQ exhibited a substitution with bulky W72, which reduced the pocket size of the StfQ N-terminal pocket. Moreover, StmE exhibited high conservation with F35, F81, and H103, which was observed in BexL rather than in StfQ. L204 and T300, which were distinguished at the entrance of the pocket defined by the loop region, also matched BexL rather than StfQ (Figure S3). These residues influence the direction of specific interactions with the respective ACPs. StmE represents dual-domain ARO/CYCs from type II polyketide synthases, similar to BexL, which are responsible for the synthesis of aromatic polyketides with a C7–C12 first-ring cyclization pattern. However, StmE and BexL differ from StfQ because they possess (StmE and BexL) or lack (StfQ) the NADPH-dependent KR. Thus, it can be postulated that StmE cyclizes the elongated polyketide chain between C7 and C12 in setomimycin biosynthesis, and subsequently reduces the C9 carbonyl to a hydroxyl group. Following aromatization by StmE, the resulting compound underwent elimination of the hydroxyl group, yielding water.

Nascent polyketide chains are cyclized by ARO/CYC to form a basic aromatic skeleton. In certain instances, the ketone groups of the polyketide chains may be subjected to selective reduction by KR, either before or after aromatization and cyclization. As structural templates, basic frameworks are important for the generation of structural diversity. Nevertheless, despite the prevalence of aromatic polyketides, only eight basic skeletons have been identified comprising the following eight types of ARO/CYCs: TcmI, TcmJ, TcmN (te-tracenomcyns), SnoaL (nogalamycin), CalC (calicheamicins), OxyN (oxytetracycline), RemFL (resistomycin), and metallo-β-lactamase (MβL)-type cyclases [36]. Moreover, these structures typically comprise successive conjugated aromatic ring scaffolds which require the addition of two to four types of ARO/CYC to form. As anticipated, the presence of two additional cyclases (StmH and StmK) in the setomimycin BGC was confirmed, exhibiting the most analogous 3D structures to TcmI (PDB: 1TUW) and AbxD (PDB: 8IS2) via HHpred and Phyre2 3D structure prediction programs [37]. StmH and StmK exhibit 34.57% identity and 52.26% similarity, suggesting that they may form dimers analogous to KS and CLF in the minimal PKS. Moreover, whereas TcmI and AbxD comprise 109 and 113 amino acids, respectively, StmH and StmK are approximately twice as long (216 and 217 amino acids, respectively). In addition, the N-terminus and C-terminus of StmH and StmK shared 27.73–39.68% identity and 46.22–59.68% similarity in amino acid sequence identity (Table S3). Subsequently, we conducted a comparative analysis of the crucial amino acids implicated in substrate specificity and catalysis, as depicted in the crystal structures of TcmI and AbxD, with the amino acid sequences of the N- and C-termini of StmH or StmK. Among the three amino acids that play a role in controlling and catalyzing the cyclization and aromatization in TcmI and AbxD, R40, Y49, and H51 are conserved in both the N- and C-termini of StmH/StmK. In contrast, D27 is conserved in the N-terminus of StmH, the C-terminus of StmH, and the N-terminus of StmK. The C-terminus of StmK was substituted with Asp (Figure S4) [37]. The peculiar protein size, amino acid composition, and mutual similarity of the N-terminus and C-terminus of StmH and StmK may lead to the expectation of additional ARO/CYCs and a unique catalytic mode of aromatic polyketide frameworks compared with other conventional TcmI-type ARO/CYCs. Finally, it is possible that StmH and StmK cyclases participate in the formation of ring-B and C, respectively, following CYC/ARO StmE, or that StmH and StmK act simultaneously to form only ring-B and yield ring-C formation to type II thi-oesterase (TEII) StmF.

#### 2.2.3. Chain Release

Subsequently, the thioester bond between ACP and the polyketide skeleton is cleaved in a process known as chain release or offloading, the mechanism of which remains unclear. Chain release is a common and important step in the biosynthesis of both polyketides and nonribosomal peptides. TE-mediated product release has been extensively investigated and is well understood. Both type I PKS and NRPS are classified as type I TEs, as most TE enzymes are typically found at the C-terminus of each integrated domain [38]. An alternative approach to chain release is to use a TEII enzyme encoded elsewhere in the BGC. Similar to the TEs of PKS-I and NRPS, TEIIs contain an α/β hydrolase fold and conserved Ser-Asp-His triad. In contrast, in type II polyketide biosynthesis, the mechanism of chain release of thioester bond breakage is also an important event, although its precise mechanism remains largely unknown [38,39,40]. In this context, a discrete TEII, AlpS, was initially identified in the BGC of kinamycin, an aromatic polyketide. Although AlpS does not function as a typical TEII, it has been demonstrated to play a pivotal role in offloading the decaketide intermediate from ACP [39]. Amino acid analysis of AlpS revealed the presence of a highly conserved GxSxG motif in TEII and a conserved catalytic triad at Ser89, Asp202, and His230. Recent studies have collectively identified the complete biosynthetic cluster responsible for the closely related metabolite setomimycin and demonstrated multiple enzymatic steps in the bisaryl preanthraquinone biosynthesis process [9,10,11,12,13,14]. Amino acid sequence alignment revealed that, among the uncharacterized genes, StmF encodes a unique enzyme belonging to the α/β hydrolase family, which is rarely observed in type II polyketide synthase (PKS) systems. Upon examination of the amino acid sequence of StmF, we identified a GHSMG motif and a Ser-Asp-His catalytic triad that are highly conserved in TEII with editing and hydrolysis functions. Further bioinformatics analysis revealed that StmF belongs to the InterPro family IPR012223, indicating its potential role in the editing process during monomeric setomimycin biosynthesis (Figure S5). Furthermore, an HHpred structure homologue search demonstrated that StmF exhibits high similarity to AntI and GrgF, which are well-known TEIIs with an editing function in polyketide Q256 and gregatin A biosynthesis, respectively [40,41,42]. Sometimes, the GxSxG motif of the initial alanine was present in lieu of a glycine residue, as observed in SpmF, JuiF, HlmB, and LinF. The rationale for the selection of a catalytic alanine over a glycine residue using TE remains unclear. Nevertheless, the presence of catalytic alanine can indicate other preanthraquinone PKSs, suggesting that TEII may exhibit unusual activity. This hypothesis will be further investigated in future studies. We propose that the role of StmF in the setomimycin biosynthesis pathway is chain release in polyketide biosynthesis.

#### 2.2.4. Dimerization

Cytochrome P450 enzymes are involved in the intermolecular oxidative phenol coupling process for the dimerization of monomers of biaryl compounds, including julichromes, spectomycins, lincolnian, and setomimycin [43,44,45,46,47,48,49]. As anticipated, the P450-like enzymes StmI, SetI, and SemI are present in setomomycin BGCs, and their corresponding enzymes have been identified in lincolnenins, julichromes, and spectomycins. Furthermore, StmI was identified by a tertiary structure prediction program search using HHpred as the enzyme responsible for the oxidative phenol coupling reaction between OxyA and OxyB (PDE, 1LFK) during vancomycin biosynthesis. AspB (PDB, 8TWU), which is involved in the dimerization of diketopiperazine (DKP), and cytochrome P-450 (PDE, 6KZS), which are involved in biaryl coupling, although the specific compound is unknown in *S. griseus*. Setomimycin P450s (StmI, SetI, and SemI) exhibit typical P450 fold characteristics, with helix L containing the signature sequence, FGHGXHXCLG (H351 substituted with Y351). In addition, the proximal axial thiolate ligand of heme iron, Cys347, is conserved. Moreover, multiple sequence alignments of putative setomimycin P450s, OxyA, OxyB, and AspB, with structurally confirmed P450s from bacteria also identified the highly conserved ^244^A/GGxxT^249^ motif characteristic of conserved P450s, the ^283^ExxR^286^ motif in the K-helix, and the ^351^GxxxC^355^ motif in the heme-binding ring [50,51]. However, the A/GGXXT motif has not been identified in biaryl polyketides other than setomimycin: lincolnenins, julichromes, and spectomycin P450. Furthermore, spectomycin P450 exhibited a substitution of the F349 residue in the FGHGxHxCLG motif with a Q349 residue. These differences may be related to the substrate specificity of the biaryl polyketides and the C-C position of the phenol coupling (Figure S6).

Ferredoxins, StmM, and cytochrome P450 (StmI) enzymes are co-located in operons, suggesting their functional relevance. The protein structure prediction programs HHpred and Phyre2 revealed a high similarity between the proteins from *Thermotoga maritima* (PDB, 1VJW), *Synechococcus elongatus* (PDB, 1JB0), *Desulfovibrio gigas* (PDB, 1FXD), and *Rhodopseudomonas palustris* (PDB, 4ID8). Furthermore, StmM is like [3Fe-4S]-type ferredoxins from *Mycobacterium tuberculosis* (PDB, 8AMP, and 8AMQ) [52,53,54,55,56]. Most Fe4S4 ferredoxins found in bacteria are ligated by four cysteine residues and share a consensus motif of CxxCxxC or CxxC (where C stands for cysteine and x stands for any amino acid) and one (or two) cysteines further apart in sequence space [57]. However, the CxxC unit was not observed in StmM or in its homologues SetM from *S. aurantiacus* JA 4570, *S. justiciae* RA-WS2 SemM, and LinM from *S. lincolnensis* NRRL 2936.

Conversely, StmM comprises 64 amino acids and contains three cysteine residues at Cys12, Cys18, and Cys56. These correspond exactly to Cys10, Cys16, and Cys54 of *M. tuberculosis* Fdx and FdxE [3Fe-4S] ferredoxins. In addition, both Fdx and FdxE contain the same CxxHxxC(x)nCP motif and proline, which are invariably conserved in all 3Fe-4S ferredoxins. StmM also contained a CxxAxxC(x)nCP motif with His substituted by Ala (Figure S7). The His15 residues of Fdx and FdxE have not been linked to the [3Fe-4S] cluster by hydrogen bonding. This observation led to the inference that StmM and its counterparts, SetM, SemM, and LinM, are [3Fe-4S]-type ferredoxins. These ferredoxins are not present in the julichrome and spectomycin BGCs, which are like setomimycin and linconenin [58]. In conclusion, based on the hypothesis that StmI and StmM play significant functional roles in setomimycin biosynthesis, we propose several directions for future research to further investigate their roles and gain a deeper understanding of the mechanisms underlying biaryl dimerization. First, it will be essential to express and purify recombinant StmI and StmM proteins and subsequently reconstruct the dimerization reaction in vitro to explore their reaction mechanisms. Second, testing a range of primary biaryl substrates, including analogs of setomimycin, will be important for determining the substrate specificity of StmI. Third, kinetic analyses should be conducted to assess the efficiency and mechanism of dimerization catalyzed by StmI in the presence of StmM. Finally, it would be prudent to consider molecular docking and dynamics simulations to predict the binding interactions between StmI, StmM, and potential substrates. These research directions are expected to provide deeper insights into the functional roles of StmI and StmM.

The final enigma in Stm biosynthesis is the presence of StmL and StmN. Amino acid sequence comparison indicated that StmL, comprising 142 amino acids, is highly similar to StmN, comprising 152 amino acids (32.95% identity and 46.82% similarity), which has the potential to form a heterodimer. The two proteins were not found in any other julichrome and streptomycin BGCs, except for setomimycin BGCs. Furthermore, it is absent from the lincolnenin BGC of *S. lincolnensis* NRRL2936, a bianthracene that is highly structurally similar to setomimycin. A simple comparison of the lincolnenins, julichromes, spectomycin, and huanglongmycin BGCs with the setomimycin BGC allowed us to speculate as follows. First, the presence or absence of StmI-like enzymes results in different dimerization patterns for setomimycin and lincolnenins, including the positioning specificity of C-C bond formation, compared to julichromes and spectomycin. Second, the exclusive presence of StmL and StmN within the setomimycin BGC may cause disparate dimerization or post-modification processes which are distinct from those observed in lincolnenins. It seems plausible to suggest that StmL and StmN are involved in setomimycin-specific dimerization or post-modification, rather than monomer formation. StmL and StmN belong to the nuclear transport factor 2 (NTF-2)-like protein family, which is exemplified by a twisted α + β barrel fold. Recent reports have demonstrated that some members of the NTF-2-like protein family commonly catalyze a range of reactions, including dehydration, dehydrochlorination, decarboxylation, and more recently, formaldehyde-eliminating lyase reactions. Some well-characterized enzymes include 3-oxo-Δ4-chenodeoxycholyl-CoA dehydratase (BaiE), scytalone dehydratase (SDH), γ-hexachlorocyclohexane dehydrochlorinase (LinA), gallate/protocatechuate decarboxylase (GDC), and erythro-DGPD γ-formaldehyde lyase (LdpA) [59,60].

A comparison of the three-dimensional structures using the HHpred structure and Phyre2 program also indicated that StmL and StmN are the most similar structural models to the three-dimensional structures of scytalone dehydratase (PDB 3EF8), LdpA lyase (PDB 8ABV), and numerous BaiE/LinA-like enzymes of unknown function. As anticipated, based on structural analysis, StmL and StmN exhibit a shared catalytic theme involving a conserved His-Asp dyad that functions as an acid–base catalyst, analogous to that observed in NTF2-like proteins [59,60]. In addition, there is a quadratic similarity to polyketide cyclases, such as SnoaL. However, the essential active site residues are unlikely to be highly conserved and, therefore, unlikely to participate in monomeric cyclization. Moreover, setomimycin has a relatively simple three-ring structure, and the setomimycin BGC contains sufficient enzymes, including aromatase (StmE), cyclase (StmH and StmK), and thioesterase (StmF). StmL and StmN exemplify the catalytic reactions of the NTF2 proteins. Their potential involvement in the release of the carboxyl group of the starter unit and the dehydrogenation reaction after dimerization represents the final challenge in setomimycin biosynthesis. The possibility of decarboxylase and dehydrogenase activity is a promising avenue for further investigation.

#### 2.2.5. Concluding Remarks

**Figure 3:**
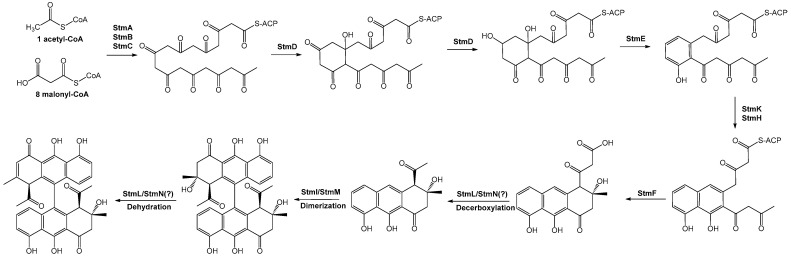
The proposed pathway for setomimycin biosynthesis in *S. nojiriensis* JCM 3382 was consistent with the data generated in this study. StmL and StmN are heterodimeric proteins with high similarity to NTP-2 family proteins. Further research is needed to elucidate their roles in setomimycin biosynthesis. The putative functions of each gene for the setomimycin BGC are shown in Table S2.

Natural nonaketide-derived aromatic polyketides, including the biaryl compound setomimycin, are biosynthesized by the Claisen-like condensation of one acetyl-CoA and eight malonyl-CoAs. Nonaketide precursors can form homo- or heterodimers via oxidative phenol coupling, a process that has garnered significant interest from enzymologists and combinatorial chemists engaged in studies aimed at elucidating the biosynthesis of these polyketides. The setomimycin biosynthetic pathway was inferred based on three main sources of information: previously identified type II polyketide BGSs, 3D structures of type II PKSs, and significantly conserved amino acid sequences from mutational studies. The initial biosynthesis of setomimycine is also conducted by the minimal PKS complex, which comprises a ketoacyl synthase (StmA), CLF (StmB), and ACP (StmC) that catalyzes the Claisen condensation of one molecule of acetyl-CoA to eight molecules of malonyl-CoA. The resulting poly-β-keto thioester nonaketide was then subjected to controlled folding by 9-ketoreductase (StmD), aromatase (StmE), second-/third-ring cyclases (StmH and/or StmK), and thioesterase (StmF) enzymes, generating the first stable monomeric setomimycin. Further reactions with P450 and ferredoxin enzymes furnished the dimeric form of the biaryl compound setomimycin (Figure 3). Proposals for Stm biosynthesis present unusual processes and biosynthetic enzymes distinct from the previously engineered biosynthesis of bacterial aromatic polyketides over the past decades. The discovery of new isomeric forms of bianthracene polyketides offers opportunities for the rational design of microbial cell factories for aromatic polyketides. Furthermore, the generation of novel isomeric bianthracenes, including “unnatural” derivatives, is a viable prospect. To further elucidate the biosynthesis of monomeric and dimeric polyketides of bianthracene, future research will focus on the role of StmH and StmK in the cyclization of the monomeric form of setomimycin, the participation of NTF-2-like proteins StmL and StmN in decarboxylation or dehydrogenation, and the relationship between dimerization patterns and specificity of ferredoxin and P450 enzymes.

### 2.3. α-Glucosidase Inhibitory Activity of Setomimycin and Streptomyces nojiriensis JCM3382

**Figure 4:**
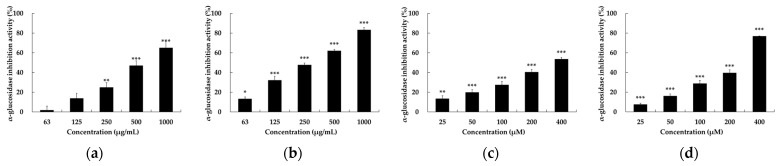
α-Glucosidase inhibition activity of different concentrations of acarbose (**a**,**c**), JCM3382 EtOAc extracts (**b**), and setomimycin (**d**) in the presence of p-NPG. JCM3382 EtOAc extracts (**b**) and setomimycin (**d**) showed lower IC_50_ than each positive control acarbose (**a**,**c**). Data are presented as the mean ± SEM. * *p* < 0.05, ** *p* < 0.01, and *** *p* < 0.001 compare with not-treated group.

Biaryl polyketides, including spectomycin and julichromes such as setomimycin, have been identified as possessing a diverse range of biological activities, including antibacterial, anticancer, and SUMOylation inhibition [11,12,13]. However, no AGI studies have been conducted on biaryl polyketides to date. The AGI activities of JCM3382 EtOAc extract and setomimycin were quantified in 96-well plates with 4-nitrophenyl β-D-glucopyranoside (p-NPG) as the substrate, in accordance with the methodology outlined in the experimental section. An equal volume of phosphate-buffered saline (PBS) was used as a negative control, whereas acarbose, a representative α-glucosidase inhibitor, was used as a positive control. Compared to acarbose, the JCM3382 EtOAc extract and setomimycin were observed to demonstrate a notable inhibitory effect on α-glucosidase, with IC_50_ values of 231.26 ± 0.41 μM and 285.14 ± 2.04 mg/mL, respectively. Setomimycin demonstrated a more pronounced inhibitory effect than the clinically used drug acarbose (Figure 4).

### 2.4. Chemoinformatic Analysis

In this study, we comprehensively analyzed the pharmacokinetic properties of setomimycin and acarbose, particularly focusing on the following parameters: molecular weight, hydrogen bond acceptors, hydrogen bond donors, rotatable bonds, topological polar surface area, Log *p*, molar refractivity, and Lipinski’s Rule of Five analysis [61] (Table 1). Setomimycin exhibits a balanced pharmacokinetic profile despite its high molecular weight (>500 Da). The compound met Lipinski’s criteria, with a TPSA of 169.43, indicating a favorable aqueous solubility. The Log *p* value (3.476) indicates a favorable absorption profile, whereas the molar refractivity (MR) value (160.66) aligns with the expected biological activity. However, acarbose exhibits a significant divergence from Lipinski’s rule, attributable to its elevated molecular weight (645.60) and a substantial number of rotatable bonds (9). This potential impact on oral bioavailability persists despite a TPSA of 90.90, which suggests reasonable hydrophilicity.

Despite a minor violation of Lipinski’s rule, setomimycin exhibits potential oral bioavailability. Acarbose exhibits multiple violations and encounters difficulties in terms of absorption. These insights will inform the optimization of compounds for therapeutic applications.

### 2.5. Docking and Molecular Dynamics (MD) Simulations

#### 2.5.1. Molecular Docking Simulation

**Figure 5:**
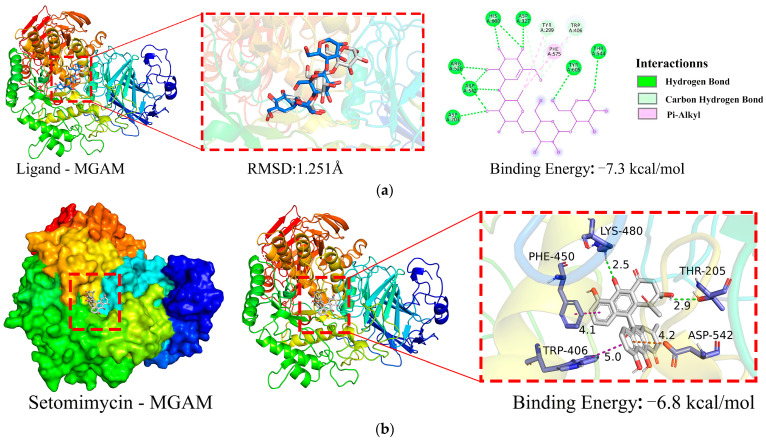
Binding interactions of MGAM protein with ligands. (**a**) MGAM–co-crystallized ligand; (**b**) MGAM–setomimycin.

Figure 5a illustrates that the co-crystallized ligand (acarbose) maintained its binding position with the MGAM protein (PDB ID: 2QMJ) with minimal differences in conformation before and after redocking, yielding a root-mean-square deviation (RMSD) value of Å (Figure 5a). This indicated successful methodological validation. A binding energy below 0 kcal·mol^−1^ indicates that the receptor and ligand can spontaneously bind, whereas a binding energy more negative than −5 kcal·mol^−1^ signifies excellent binding affinity. The binding energy between the co-crystallized ligand and MGAM is −7.3 kcal·mol^−1^, indicating a strong binding interaction between the ligand and protein.

The docking binding energy between MGAM protein and setomimycin was found to be −6.8 kcal·mol;^−1^, indicating a high binding affinity (Figure 5b). The two hydroxyl groups on setomimycin form hydrogen bonds (green dashed lines) with MGAM chain A residues THR-205 (2.9 Å) and LYS-480 (2.5 Å), π–π interactions (purple dashed lines) with TRP-406 (5.0 Å) and PHE-450 (4.1 Å), and a π–cation interaction (orange dashed line) with ASP-542 (4.2 Å). The results of 10 repeated docking experiments demonstrated high reproducibility, with the binding energy varying within ±0.1 kcal·mol^−1^. Setomimycin binds to the same active pocket in the MGAM protein as the co-crystallized ligand, with a binding energy of −6.8 kcal·mol^−1^, indicating high affinity and may have potential pharmacological effects on MGAM’s structure and function.

#### 2.5.2. Molecular Dynamics (MD) Simulation

**Figure 6:**
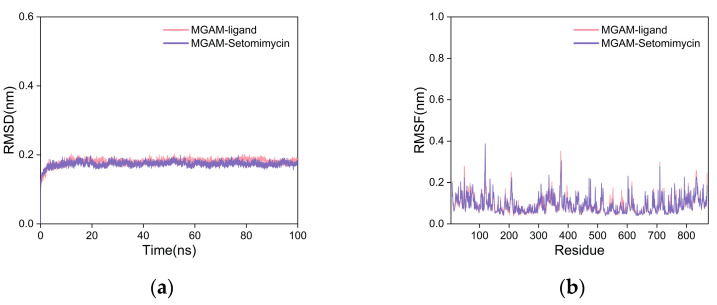
Analysis of the MD simulation results. (**a**) RMSD and (**b**) RMSF curves.

To further assess the binding stability and affinity, we performed 100-nanosecond MD simulations of the complexes of the co-crystallized ligand, setomimycin, and MGAM. This was done because the semiflexible docking method does not account for protein flexibility. The smaller RMSD values observed on the curve indicate stable protein–ligand complexes with minimal structural changes. The RMSD curves for the MGAM protein complexes with the co-crystallized ligand and setomimycin remained within 0.2 nm throughout the simulation, indicating minimal fluctuations (Figure 6a). The two curves exhibited substantial overlap, indicating that the MGAM–setomimycin complex exhibited high stability comparable to that of the co-crystallized ligand complex.

The root-mean-square fluctuation (RMSF) curve quantifies fluctuation of individual amino acid residues during dynamics. Higher values indicated more fluctuation, whereas lower values indicated less fluctuation. The RMSF curves for both complexes exhibited nearly identical fluctuations, with values within 0.4 nm and no significant deviations (Figure 6b). This indicated that the addition of either the co-crystallized ligand or setomimycin had a minimal impact on the stability of the amino acid residues in the MGAM protein, suggesting that both complexes exhibit good stability.

**Figure 7:**
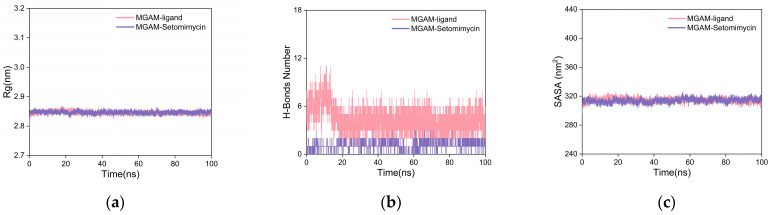
Analysis of the MD simulation results. (**a**) Rg curves; (**b**) H-bond plot; (**c**) SASA plot.

The radius of gyration (Rg) is a measure of structural compactness and stability of a molecule. A larger value indicates expansion, whereas a smaller value indicates compactness and stability. As illustrated in Figure 7a, the Rg curves for the MGAM protein complexes with the co-crystallized ligand and setomimycin exhibit near-overlapping patterns, with fluctuations within the 2.8–2.9 nm range and no significant deviations. This suggests that both the co-crystallized ligand and setomimycin formed compact and stable complexes with the MGAM protein.

To investigate the role of hydrogen bonding at the binding sites, we calculated the key hydrogen bonds (H-bonds) that stabilize the ligand–protein interactions in the complexes. Figure 7b shows that after 20 ns, the hydrogen bond curves between the MGAM protein and the co-crystallized ligand or setomimycin exhibited stabilization. The co-crystallized ligand maintained between three and five hydrogen bonds, indicating strong and stable interactions. Setomimycin formed one to two stable hydrogen bonds with the MGAM protein, suggesting stable but fewer interactions compared to the co-crystallized ligand, implying a slightly weaker binding affinity.

Solvent-accessible surface area (SASA) was used to elucidate protein folding and stability. Stable proteins exhibited more consistent SASA curves. The SASA curves for both complexes in Figure 7c exhibited nearly overlapping, consistent, and stable fluctuations throughout the simulation, indicating that the MGAM protein complexes with the co-crystallized ligand and setomimycin had stable structures.

**Figure 8:**
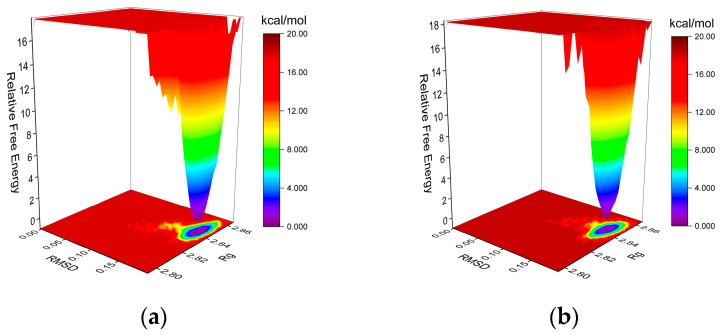
The Gibbs FEL plots. (**a**) MGAM–co-crystallized ligand; (**b**) MGAM–setomimycin.

The free-energy landscape (FEL) is generated via Gromacs scripts, specifically g_sham and xpm2txt.py, which facilitate the computation of Gibbs free energy in relation to RMSD and Rg. The plot integrates RMSD, Rg, and Gibbs free energy on the X-, Y-, and Z-axes, respectively, illustrating the energetically favored conformations that emerge during complex dynamics. Weak interactions result in the formation of multiple rough energy clusters, whereas strong interactions yield a single smooth cluster. The dark purple and blue spots indicate energy minima, representing stable conformations, whereas the red and yellow spots denote unstable conformations. Both MGAM protein complexes with the co-crystallized ligand (Figure 8a) and setomimycin (Figure 8b) exhibited a single centralized energy cluster on the FEL plot, indicating their stability.

**Figure 9:**
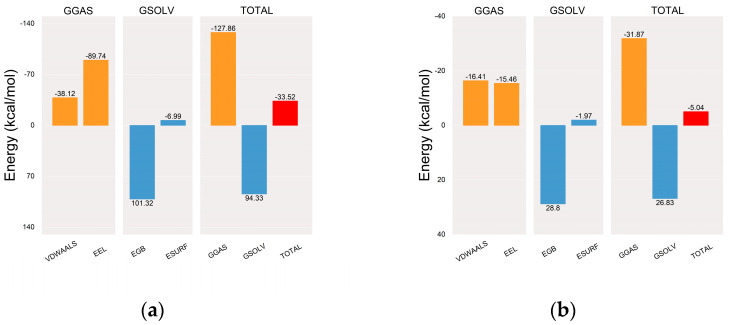
MM-PBSA binding energy plots. (**a**) MGAM–co-crystallized ligand; (**b**) MGAM–setomimycin. VDWAALS, EEL, EGB, ESURF, GGAS, GSOLV, and TOTAL denote specific energy terms in MD simulations: van der Waals interactions, electrostatic energy, polar solvation energy, nonpolar solvation energy, molecular mechanics, solvation energy, and average binding free energy, respectively.

Once equilibrium was reached, MM/PBSA binding free energies were calculated for complexes of the MGAM protein with the co-crystallized ligand (Figure 9a) and setomimycin (Figure 9b). The mean binding free energies were −33.52 and −5.04 kcal·mol^−1^, respectively, indicating a robust affinity between the MGAM protein and the co-crystallized ligand. Notwithstanding the potential for multiple rigid cyclic structures in setomimycin to reduce its affinity, 100 ns MD simulations demonstrated the formation of stable complexes with the MGAM protein, comparable to those observed with the co-crystallized ligand.

**Figure 10:**
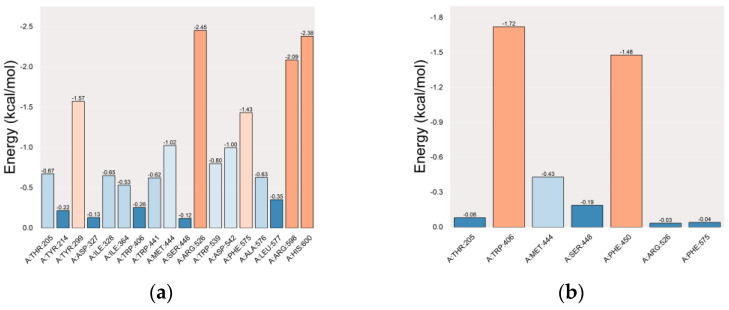
Residue-energy plots. (**a**) MGAM ligand; (**b**) MGAM–setomimycin.

In residue-energy analysis, the co-crystallized ligand interacts with the MGAM protein residues ARG-526, HIS-600, and ARG-598 with high binding energies of −2.45, −2.38, and −2.09 kcal·mol^−1^, respectively. This highlights the significant role of these residues in binding (Figure 10a). Setomimycin exhibits the strongest interactions with MGAM residues TRP-406 and PHE-450, with binding energies of −1.72 and −1.48 kcal·mol^−1^, respectively. These findings indicated that these residues play a crucial role in the binding of setomimycin to the MGAM protein (Figure 10b).

**Figure 11:**
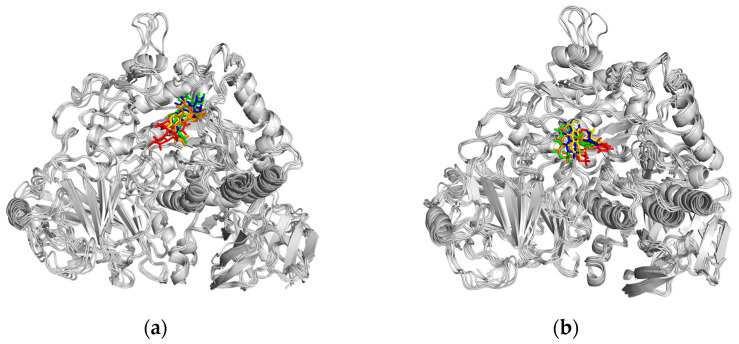
Conformation plots of the complexes. (**a**) MGAM ligand; (**b**) MGAM–setomimycin. The configurations at different time points are represented as follows: 0 ns (green), 25 ns (blue), 50 ns (brown), 75 ns (yellow), and 100 ns (red).

Binding stability was evaluated by comparing the complex conformations at five time points (0, 25, 50, 75, and 100 ns) in MD simulations. Both the co-crystallized ligand (Figure 11a) and setomimycin (Figure 11b) consistently bound to the MGAM protein without significant positional changes, thereby demonstrating the excellent stability of the complexes.

## 3. Materials and Methods

### 3.1. Chemicals and Reagents

Setomimycin was procured from Santa Cruz Biotechnology (Dallas, TX, USA). The α-glucosidase enzyme from the yeast *Saccharomyces cerevisiae* (750 units), p-NPG, and acarbose were procured from Sigma-Aldrich (St. Louis, MO, USA). Yeast extract and malt extract (ISP2) medium were obtained from KisanBio (Seoul, Republic of Korea). All other commercial solvents, chemicals, and reagents were purchased from Sigma-Aldrich (St. Louis, MO, USA) and had the highest purity. The samples were used without further purification.

### 3.2. Fermentation and Extraction

*S. nojiriensis* JCM 3382 was procured from the Japan Collection of Microorganisms (JCM) of the Microbe Division in RIKEN-BRC and maintained in ISP2 medium (yeast extract 0.4%, malt extract 1%, dextrose 0.4%, and agar 20%, pH 7.2). Culture seeding of the strain was conducted in ISP2 broth for 2 days. In the scale-up fermentation process, the seed cultures, which were grown in ISP2 medium (50 mL), were transferred aseptically to 5 L Erlenmeyer flasks containing 1 L YM medium. These cultures were then incubated at 30 °C in a rotary shaker (200 rpm) for 7 days. Following a 7-day cultivation period, 1 L ISP2 broth was centrifuged at 4000 rpm for 20 min. The resulting supernatant was extracted with EtOAc (3 × 1 L) and the combined extracts were concentrated under reduced pressure.

### 3.3. Enzymatic Analysis

The α-glucosidase inhibitory activities of the *S. nojiriensis* EtOAc extract, setomimycin, and acarbose were quantified using the following methodology: First, a stock solution of 1.5 mM p-NPG, 750 mU/mL α-glucosidases, 4 mM setomimycin, and 4 mM acarbose was prepared using 0.1 M PBS buffer (pH 7.4). The experiment was conducted in 96-well plates with a total volume of 200 μL. In summary, 20 μL of each setomimycin and acarbose solution were added to a 96-well plate, followed by 100 μL of p-NPG. Subsequently, 20 μL of α-glucosidase was added to each well, and the plates were incubated at 37 °C for 10 min. Subsequently, the reaction was terminated by the addition of 60 μL of 0.1 M Na_2_CO_3_. Optical density (OD) was measured at an absorbance wavelength of 405 nm using a microplate reader (Tecan, Switzerland). The α-glucosidase inhibitory activity was calculated using the following equation:

(1) $$\alpha \text{-}Glucosidaseinhibition\;activity\;(\%)=(\frac{\Delta A_{\mathrm{control}}-\Delta A_{\mathrm{sample}}}{\Delta A_{\mathrm{control}}})\times 100$$

The IC_50_ value was defined as the concentration of the tested compounds that inhibited 50% of enzyme activity. The IC_50_ values were calculated from linear regression analyses of five concentrations of the tested sample using GraphPad Prism 9.0 software (GraphPad Software, La Jolla, CA, USA).

### 3.4. Chemoinformatic Analysis

Pharmacokinetic parameters were evaluated by analyzing the chemoinformatic properties derived from SMILES information using prediction websites. A comprehensive investigation was conducted using a variety of models, including ADMETlab 3.0 and SwissADME (http://www.swissadme.ch/, accessed on 8 July 2024).

### 3.5. Molecular Docking Simulation Analysis

The receptor protein, maltase-glucoamylase (MGAM), with PDB ID: 2QMJ, was obtained from the Protein Data Bank (PDB) database, and its 3D structure file was downloaded. The protein structure was examined using PyMOL version 3.0.3 to prepare for docking studies. The ligand setomimycin (PubChem CID: 198523) was obtained from the PubChem database, and its 3D structure file was downloaded. Subsequently, the ligand structure was optimized using the MMFF94 force field with the OpenBabel software. The process involved generating the 3D structure, adding hydrogen atoms, performing energy minimization, and verifying stereogenic centers using specific commands, resulting in the lowest energy and most stable conformation of the molecule.

AutoDock Tools version 1.5.6 was employed to add hydrogen to the protein and the ligand, as well as to ascertain the rotatable bonds for the ligand. The docking grid parameters were set based on the co-crystalized ligand position in the MGAM protein structure, with the center at (X, Y, Z) = (−20.4, −6.2, −2.6) and the size of the grid box being (28.0 × 28.0 × 28.0). Docking was performed using a semiflexible approach with an exhaustive value of 25 to enhance the precision and reliability of the results, and the Lamarckian genetic algorithm was employed as the docking algorithm. Molecular docking was conducted using AutoDock Vina version 1.2.0, resulting in binding free-energy values and docking result files.

To validate the reliability of molecular docking, the protein and ligands were subjected to 10 repeated dockings under the same conditions, and the differences between the results were compared. Furthermore, redocking was conducted using the co-crystallized ligand from the protein structure, and the RMSD values before and after docking were calculated. An RMSD value of less than 2 Å indicated successful methodological validation [62,63].

### 3.6. Molecular Dynamics (MD) Simulations Analysis

MD simulations of the MGAM protein complexes with the co-crystallized ligand and setomimycin were conducted using Gromacs 2022 [64]. The Amber14sb force field was selected for the protein and Gaff2 was employed as the ligand. The SPC/E water model was employed to solvate the protein–ligand systems in a periodic boundary box of 1.2 nm. Long-range electrostatic interactions were calculated using the Particle Mesh Ewald (PME) method, and appropriate numbers of sodium and chloride ions were added to neutralize the system using the Monte Carlo method. Before the commencement of the main simulation, the system underwent energy minimization and equilibration in three steps. First, the system was subjected to 50,000 steps of steepest descent energy minimization, and the process was terminated when the maximum force reached a value of less than 1000 kJ/mol. Subsequently, NVT pre-equilibration with a constant number of particles, volume, and temperature (310 K) was conducted for 50,000 steps with a 2 fs time step. Subsequently, NPT pre-equilibration was performed with a constant number of particles, pressure (1 atm), and temperature (310 K) for 50,000 steps with a 2 fs time step. After the completion of the energy minimization and equilibration procedures, a 100 ns molecular dynamics simulation was conducted without constraints, using a time step of 2 fs and saving the coordinates at 10 ps intervals.

### 3.7. Statistical Analyses

All statistical analyses were conducted using GraphPad Prism v6.0 (GraphPad Prism version 6.00 for Windows, GraphPad Software, La Jolla, CA, USA). Error bars represent the standard deviation (S.D.), and a *p* value of less than 0.05 was considered statistically significant. All in vitro experiments were conducted in triplicates.

## 4. Conclusions

Biaryl polyketide setomimycins represent a unique class of antibiotics isolated from actinobacteria, particularly *S. nojiriensis* JCM 3382, *S. aurantiacus* JA 4570, and *S. justiciae* RA-WS2. The monomeric subunits of setomimycin, julichromes, lincolnenins, and spectomycins are typically derived from common polyketide precursors. Comparison of their BGCs revealed that they all had minimal PKS (StmA to StmC), C-9 ketoreductase (StmD), aromatase (StmE), and thioesterase (StmF) in the same orientation and configuration. However, compared to other biaryl preanthraquinone BGCs, the setomimycin BGC exhibits a distinctive array of biosynthetic enzymes involved in cyclization, dimerization, and post-PKS modification. Coupling of the two monomeric subunits occurs in a regioselective manner, contingent on the biaryl preanthraquinone-producing strains. In this phenol coupling, P450s play a pivotal role, and we confirmed the presence of a P450 (StmI) in the setomimycin BGC. Uniquely, in the setomimycin BGC, ferredoxin (StmM), a putative [3Fe-4S] type, is present in translational coupling with StmI. In this study, we identified the need for further investigation into the role of heterodimeric StmH and StmK. Besides their unusual size compared to classical TcmI-like cyclases, these proteins also show homology at their respective N- and C-termini. Furthermore, StmL and StmN, NTF-2-like proteins present only in the setomimycin BGC, warrant further investigation.

Biaryl polyketides isolated from bacteria have been reported to exhibit antimicrobial, anticancer, and other bioactivities. However, they have been found to have limited anti-α-glucosidase activity. Fortunately, setomimycin demonstrated 1.4-fold higher inhibitory activity against α-glucosidase with IC_50_ values of 231.26 ± 0.41 µM when compared with the available marked drug acarbose (IC_50_ = 331.32 ± 1.35).

Setomimycin, despite a minor deviation from Lipinski’s rule, shows promise for oral bioavailability, whereas acarbose, due to multiple violations, presents significant absorption challenges.

Setomimycin exhibits a strong binding affinity for the MGAM protein, with a docking energy of −6.8 kcal·mol^−1^. This interaction is characterized by the formation of crucial hydrogen bonds and π–π interactions, which are analogous to those observed for the co-crystallized ligand. The stable RMSD, RMSF, Rg, SASA, and H-bond profiles, despite a reduction in the number of H-bonds, indicated significant structural stability and potential pharmacological impact in MGAM-related studies. These findings provide new insights and valuable information for the study of the mechanism of inhibition of α-glucosidase by setomimycin, which represents a novel approach for biaryl polyketides. Further studies on adipocytes and in vivo experiments are necessary to demonstrate the antidiabetic activity and mechanism of action of this compound.

## Figures and Tables

**Table 1:** Chemoinformatic properties of compounds.

Compounds	MW	HBA	HBD	RB	TPSA	Log *p*	MR	Lipinski’s Rule
Setomimycin	580.58	9	5	3	169.43	3.476	160.66	Yes; 1 violation: MW > 500
Acarbose	645.60	5	3	9	90.90	3.307	136.69	No; 3 violations: MW > 500, N or O > 10, NH or OH > 5

MW: molecular weight (g/mol); HBA: num. H-Bond acceptors; HBD: num. H-Bond donors; RB: num. rotatable bonds; TPSA: topological polar surface area (Å2); MR: molar refractivity.

## Data Availability

Data are contained in the article and Supplementary Material.

## Supplementary Materials

The following supporting information can be downloaded at: https://www.mdpi.com/article/10.3390/ijms251910758/s1.
