# Supplementary material for: Analysis of the Setomimycin Biosynthetic Gene Cluster from Streptomyces nojiriensis JCM3382 and Evaluation of Its α-Glucosidase Inhibitory Activity Using Molecular Docking and Molecular Dynamics Simulations
Source: Int J Mol Sci. 2024 Oct 6;25(19):10758. doi: 10.3390/ijms251910758 (PMC11476836; doi:10.3390/ijms251910758)
Supplement: Supplementary file 1 [file ijms-25-10758-s001.zip › ijms-3211658-supplementary.pdf]

Analysis of the Setomimycin Biosynthetic Gene Cluster from *Streptomyces nojiriensis* JCM3382 and Evaluation of Its α-Glucosidase Inhibitory Activity Using Molecular Docking and Molecular Dynamics Stimulations

Kyung-A Hyun, Xuhui Liang, Yang Xu, Seung-Young Kim, Kyung-Hwan Boo, Jin-Soo Park, Won-Jae Chi and Chang-Gu Hyun

CONTENTS

Table S1. Putative *S. nojiriensis* JCM 3382 BGCs 2

Table S2. Genetic organization of the setomimycin BGCs 4

Table S3. Comparison of amino acid homology between the N- and C-termini of StmH and StmK 5

Figure S1. Phylogenetic analysis of a cluster of 42 known KSβ genes in Type II polyketide BGCs 6

Figure S2. Sequence alignment among various type II PKS KRs 7

Figure S3. Sequence alignment of StmE and polyketide ARO/CYCs 8

Figure S4. Comaprision of amino acid sequences of TcmI, AbxD, StmH, and StmK proteins 9

Figure S5. Sequence alignment of AlpS and biaryl polyketide type II TEs 10

Figure S6. Sequence alignment of P450 eznymes 12

Figure S7. Sequence alignment of FdxE and biaryl polyketide ferredoxins 15

Table S1. Putative *S. nojiriensis* JCM 3382 BGCs*.

Regions	Type	From	To	Most similar known cluster		Similarity
Region 1	Terpene	40,991	61,998	Avermitilol	Terpene	100%
Region 2	Lanthipeptide-class-iv PKS-like Butyrolactone	119,303	167,470	Streptocollin	RiPP:Lanthipeptide	100%
Region 3	Thiopeptide LAP	168,443	197,954	Lactazole	RiPP:Thiopeptide	55%
Region 4	NRPS-like NRPS	432,526	479,497	Antipain	NRP	100%
Region 5	T2PKS	1,066,919	1,139,413	Spore pigment	Polyketide	58%
Region 6	NRPS/NRPS-like	1,561,900	1,631,094	Tambromycin	NRP	92%
Region 7	NRPS/T1PKS	1,796,196	1,861,151	Coelichelin	NRP	72%
Region 8	Butyrolactone	1,961,621	1,972,592	Neocarzinostatin	Iterative type I + Enediyne type I polyketide	4%
Region 9	NI-Siderophore	3,622,602	3,652,383	Desferrioxamin B/E	Other	100%
Region 10	T2PKS	4,276,901	4,349,362	Setomimycin	Polyketide	100%
Region 11	RiPP-like	4,584,818	4,595,105			
Region 12	NRPS-like/T1PKS/NRPS	5,057,273	5,117,610	Bleomycin A2/B2	NRP + Polyketide + Saccharide	7%
Region 13	CDPS	6,208,634	6,229,392	BD-12	NRP	17%
Region 14	NI-Siderophore	6,607,990	6,640,997	Kinamycin	Polyketide	13%
Region 15	Hydrogen-cyanide	6,812,909	6,825,953	Aborycin	RiPP	21%
Region 16	RiPP-like	6,891,612	6,902,943			
Region 17	Terpene	7,011,883	7,034,099	Toxoflavin Fervenulin	Other	14%
Region 18	Lanthipeptide-class-iv	7,095,057	7,117,990			
Region 19	Terpene	7,404,584	7,431,728	Hopene	Terpene	61%

Region 20	T1PKS/HglE-KS	7,504,501	7,555,916	Hexacosalactone A	Other	9%
Region 21	NRPS/NRPS-like	7,604,900	7,720,913	Leupeptin Pr/Ac	NRP	100%
Region 22	T1PKS	7,832,794	8,024,104	Linearmycin A-C	Type I polyketide	100%
Region 23	Lanthipeptide-class-iv	8,122,015	8,144,813			
Region 24	NRPS-like	8,186,135	8,229,356	Lipstatin	NRP	42%
Region 25	Terpene	8,288,028	8,309,149	Bombyxamycin A/ B	Polyketide	3%
Region 26	Terpene	8,385,926	8,407,281	2-Methylisoborneol	Terpene	100%
Region 27	Terpene	8,477,920	8,498,825	Monensin	Polyketide	5%
Region 28	Melanin	8,514,987	8,542,302	Istamycin	Saccharide	4%
Region 29	NI-Siderophore	8,627,590	8,658,781	Gausemycin A/B	NRP + Saccharide	4%
Region 30	T3PKS	8,688,189	8,729,250	Alkylresorcinol	Polyketide	100%
Region 31	CDPS/NAPAA	8,765,178	8,817,045	ε-Poly-L-lysine	NRP	100%

*Predicted biosynthetic gene clusters of *S. nojiriensis* JCM 3382 (antiSMASH version 7.1.0).

Table S2. Genetic organization of the setomimycin BGCs*.

S. nojiriensis JCM3382			*S. aurantiacus* JA4570 (GenBank/Identities/Positives/Gaps)	*S. justiciae* RA-WS2 (GenBank/Identities/Positives/Gaps)
QTI46191.1	StmR (199aa)	TetR family transcriptional regulator	EPH44807.1/90%/92%/0%	MCW8383185.1/87%/93%/0%
QTI46192.1	StmP (206aa)	Hypothetical protein		
QTI46193.1	StmJ (504aa)	Drug resistance transporter	EPH44808.1/92%/95%/0%	MCW8383184.1/87%/93%/0%
QTI46194.1	StmK (217aa)	Type II polyketide cyclase (TcmI)	EPH44809.1/75%/86%/0%	MCW8383183.1/69%/83%/0%
				MCW8383182.1/58%/66%/6% (StmP-like)
QTI46195.1	StmL (142aa)	NTP-2 family protein	EPH44810.1/76%/86%/0%	MCW8383181.1/79%/89%/0%
QTI46196.1	StmG (268aa)	Sensory transduction protein(regX3)	EPH44811.1/87%/92%/0%	MCW8383180.1/79%/87%/0%
QTI46197.1	StmI (406aa)	Cytochrome P450	EPH44812.1/97%/99%/0%	MCW8383179.1/92%/95%/0%
QTI46198.1	StmM (64aa)	[3Fe–4S]-like ferredoxin	EPH44813.1/89%/95%/0%	MCW8383178.1/77%/90%/0%
QTI46199.1	StmN (152aa)	NTP-2 family protein	EPH44814.1/86%/91%/0%	MCW8383177.1/76%/83%/0%
QTI46200.1	StmH (216aa)	Type II polyketide cyclase (TcmI)	EPH44815.1/85%/90%/0%	MCW8383176.1/66%/75%/9%
QTI46201.1	StmA (423aa)	β-ketoacyl synthase (KSα)	EPH44816.1/94%/97%/0%	MCW8383175.1/94%/97%/0%
QTI46202.1	StmB (397aa)	Chain length factors (KSβ)	EPH44817.1/93%/96%/0%	MCW8383174.1/90%/94%/0%
QTI46203.1	StmC (88aa)	Acyl carrier protein	EPH44818.1/86%/92%/0%	MCW8383173.1/86%/90%/3%
QTI46204.1	StmD (261aa)	C9 Ketoreductase	EPH44819.1/98%/99%/0%	MCW8383172.1/92%/95%/0%
QTI46205.1	StmE (317aa)	Aromatase/cyclase	EPH44820.1/88%/93%/1%	MCW8383171.1/79%/87%/0%
QTI46206.1	StmF (299aa)	α/β Fold hydrolase	EPH44821.1/85%/90%/1%	MCW8383170.1/79%/85%/0%
QTI46207.1	StmO (63aa)	Hypothetical protein	EPH44822.1/57%/62%/0%	
QTI46208.1	ORF1 (292aa)	4'-phosphopantetheinyl transferase superfamily protein		
QTI46209.1	ORF2 (199aa)	Phosphoribosylanthranilate isomerase		

*Genetic organization, putative functions, and amino acid homology of the setomimycin BGCs from *S. nojiriensis* JCM 3382, *S. aurantiacus* JA4570, and *S. justiciae* RA-WS2.

Table S3. Comparison of amino acid homology between the N- and C-termini of StmH and StmK.

	StmH-N (Identity/Similarity)	StmH-C (Identity/Similarity)	StmK-N (Identity/Similarity)	StmK-C (Identity/Similarity)
StmH-N	-	-	-	-
StmH-C	39.68%/52.38%	-	-	-
StmK-N	33.86%/48.82%	37.90%/59.68%	-	-
StmK-C	27.73%/46.22%	34.75%/55.93%	33.33%/51.22%	-

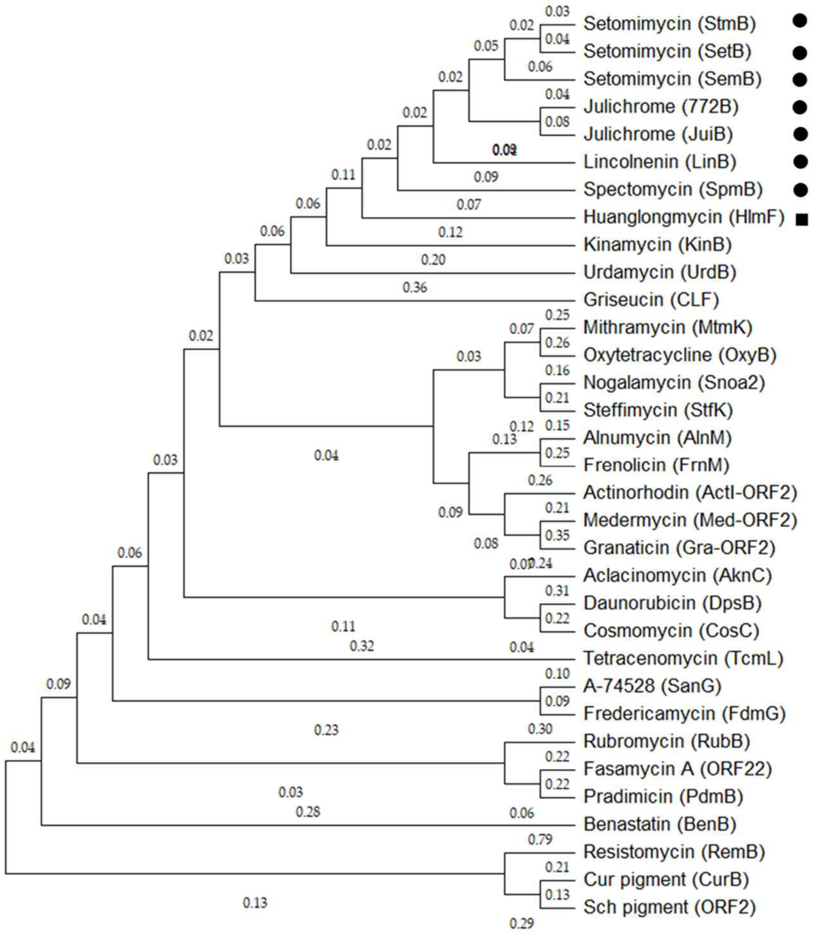

Figure S1. Phylogenetic analysis of a cluster of 42 known KSβ genes in Type II polyketide BGCs. The alignment was created by use of ClustalX and edited manually. The phylogenetic tree was constructed using MEGA11 with the method of maximum likelihood. It indicates that the putative KSβ gene StmB is located in the KSβ group for biaryl nonaketide (●) and huanglongmycin (▪) biosynthesis. Sequences included StmB (QTI46202.1), SetB (EPH44817.1), SemB (MCW8383174.1), LinB (ANS69118.1), SpmB (MCI3903331.1), JuiB (QNL10614.1), 772B (EPJ36720.1), and HlmF (AXL88815.1).

```
ActIII   MATQDSEVALVTGATSGIGLEIARRLGKEGLRVFVCARGEEGLRTTLKELREAGVEADGRTCDVRSVPEIEALVAAVVERYGPVDVLV NNAGRPGGGATAELADELWLDVVETNLTGVFR   120
HedA     --MSRPQTAFVTGVSSGIGLAVARTLAARGIAVYGCARDAKNVSAAVDGLRAAGHDVDGSSCDVTSTDEVHAAVAAAVERFGPIGILV NSAGRNGGGETADLDDALWADVLDTNLTGVFR   118
StmD     MTQQEQRVALVTGATSGIGLAVTRLLAGQGHRVFIGSRTEDKVLTTVKQLQEEQLDVDGTTCDVRSVPEIEALVAAVVERYGPVDVLV NNAGRPGGGATAELADELWLDVVETNLTGVFR   120
BexD     MTDQEGRTAVVTGATSGIGLAVSKLLARRGLRVFLGARTAENVATTVKELRDAGFEADGAACDVRSGEDVTAFVRAAVDRYGPVGVLV NNAGRPGGGVTAEIADELWLDVIETNLTSVFR   120
                    TGxxxGxG                                                                    NNAG PGGxxT

ActIII   VTKQVLKAGGMLERGTGRIVNIASTGGKQGVVHAAPYSASKHGVVGFTKALGLELARTGITVNAVCPGFVETPMAASVREHYSDIWEVSTEEAFDRITARVPIGRYVQPSEVAEMVAYLI   240
HedA     VTREVLRAGGMREAGWGRIVNIASTGGKQGVMYAAPYTASKHGVVGFTKSVGFELAKTGITVNAVCPGYVETPMAERVREGYARHWGVTEQEVHERFNAKIPLGRYSTPEEVAGLVGYLV   238
StmD     LTREVLTTGGLRDKSWGRVINIASTAGKQGVVLGAPYSASKHGVVGFTKALGNELAPTGITVNAVCPGYVETPMAERVRQNYSRLSGAPESAILEKFQAKIPLGRYSTPEEVAGLVGYLA   240
BexD     VTREVLTTGGMVAAGHGRIVNIASTAGKQGVVLGAPYSASKHGVVGFTKALGNELAPAGITVNAVCPGYVETPMAERVRQGYAAAYGTSEDEVLKKFQAKIPLGRYSTPDEVAGLVGYLV   240
                                             YxxxK

ActIII   GPGAAAVTAQALNVCGGLGNY   261
HedA     TDAAASITAQALNVCGGLGNY   259
StmD     SDTAASITSQALNVCGGLGNF   261
BexD     TDTAGSITAQALNVCGGLGNF   261
```

Figure S2. Sequence alignment among various type II PKS KRs. Sequences included hedamycin (HedA), actinorhodin (ActIII), BE-7585A (BexD), and setomimycin (StmD) KRs. Multiple-sequence alignment was performed using Clustal Omega. The SDR proteins motif (TGxxxGxG, PGxxxT, NNAG and YxxxK) were shown in red.

```
StfQ  MPTLGKDTLQYTATVAAPAPLVFDLVAGLENWPQFHGPSVHAEPLGRRDGAEEFQHWWVIDDRTVRTWRARWRFDSQALRIGYTYDPAEPAAPAQHGEWTFRRLSDASTEVRVEYEL—L  118
StmE  MPSE-LRTSEHEIKIAGPAENVYRLLAQAEKWPQLFPPNIYVDHVERGEREERIRI-WATAGDTVKHWTSHRTLDPDKLRIGFRQEVSTHPVAAMGGSWQVEALPSGETLVRLSHDFRAA  118
BexL  -----MREAEHDILVDAPADEVYRLVAEVANWPRIFPPTVFVDHVERGEGTERIRI-WATANGEPKNWTSRRELDPAARRISFHQEVSTPPVAEMSGTWIVEPVSAATAKVRLLHAYRAV  114
                   ::   : .**  *: *:*   :**::. *.:..: : * :  *.::  *.  .   : * ::  :*    **.:  : :   .    * * .. :  . : **: :

StfQ  GDEGDA-AL----ADRELRELVDCVT-DAAERHEERRDLVVDFEDPLFVAGAVDDAYTYLYEADKWPERIPHVARLVMEERVP NIQFFDMDTTTPDGSAHTTRSVRVCLPGDKIVYKQIQ  232
StmE  DD—DSLTWLEEVVDRNSRSELDSLKALAESRDAAADELTCSFEDTVQIRGAAQDVYDFIYEANLWADRLPHVITVRLDEPTPGVQT LEMDTLSKDGSSHTTKSHRVGLAPHRIAYKQTT  236
BexL  GDDPGGLAWIDRAVDTNSRSELAALKHNVEL —VTNPELTFSFTDTVRIDAPAKDVYDFVDQAALWAERLPHVSSVDLREPSPGLQV LRMDTRAKDGSVHTTESVRVCFPHHRIVYKQTT  232
      .*  .. :    .* : *. : .:.  .         :*. .* * : : . ..*.* :: :*  * :*:***  : : *  *.:* : *** : *** ***.* ** :  .:*.***

StfQ  LPKLLTGHTGHWKFTPTREGFV-LGARHTATIKP-SALPILGEGTTVLDARKYLRRVLSANSMGNLRLAKAFAEERAGV---  309
StmE  LPALITLHTGHWTFTPNDD-GVAASSQHTFTVNTDNIAKVLGADATVQDAIAYVRTALSTNSKATLGIAKEYAEGLAGAGRS  317
BexL  LPALMTLHTGRWDFAEEPGGGTTASSEHTVVLNTANIAKVLGAGAGVAEAREFVRTALSTNSRATLGFAKDHAEARP-----  309
      ** *:* ***:* *:        .  .:.** .::  .    :** .: * :*  ::* .**:** ..* :** .**
```

Figure S3. Sequence alignment of StmE and polyketide ARO/CYCs. StmE is extremely conserved with R70 in the monodomain ARO/CYCs TcmN, WhiE, and ZhuI, whereas StfQ is substituted with a bulky W72. StmE is also highly conserved with F35, F81, and H103, which are seen in BexL rather than StfQ

(a)

```
TcmI     MAYRALMVLRMDPAD-AEHVAAAFAEHDT-TELPLEIGVRRRVLFRFHDLYMHLIEADD-DIMERLYQARSHPLFQEVNERVGQYLTPYA-QDWEELKDSKAEVFYSWTAPDS----- 109
AbxD     -MATTLIVARLKPGDHRDQISRLFAESDT-TELPDLVGVQERRLLTFKDLYFHLVRTDE-ALSKTLTPQHDHPLFRSISEAMDEYVTPYE-GAWGSVEQASARQFYHWKRGLGRVQP- 113
StmK-C   -TGTVVTITRMDQAV-IPAVTSIFREFES-AESSRPTGIRRRQLFAFKDLCIHIQEYTGSNGADIAQQVASDIWSGKVVRDLPEL-APEQ-----PAANGPASRFYGWEAS------- 102
StmH-N   -MHSSVIVGRIKPGS-TEAVAGVFAGRDG--EPSGTRNLLRRQLFEYKGIYIHLQDHTQENLTEVVEHGS--------DEAFTPFLDAYDPVAGTAPSGQVATRFYEWHGQPVSDEHR 106
StmK-N   -MRTMVTVAKMDQNS-IPEVSKLFAEFDS-TEIPNMIGLRRRQLFIHDGIQIHLYDFAEKSDQAALEKVKTDPRMGRLAIDMKAFVTPYEPETWNSPADSTAFCFHDWEGEPIENPGP 115
StmH-C   -MYSTVIVARLNPAD-IGTVAKLFGDFDTGTELPHTMGTRRRQLFAYNGLYFHIQDFESDNGGELIERAKSDPRFVQIQQDLLPYISVLDPETWRSPSDAMASRFYTWQVTA ------ 110
                 : : ::.        ::  *   :    *     .  .* *: ...:  :*:                     .                     *   *: *
```

(b)

```
StmK-C   TGTVVTITRMDQAVIPAVTSIFREFES-AESSRPTGIRRRQLFAFKDLCIHIQEYTGSNG   59
StmH-N   MHSSVIVGRIKPGSTEAVAGVFAGRDG—EPSGTRNLLRRQLFEYKGIYIHLQDHTQENL   58
StmH-C   MYSTVIVARLNPADIGTVAKLFGDFDTGTELPHTMGTRRRQLFAYNGLYFHIQDFESDNG   60
StmK-N   MRTMVTVAKMDQNSIPEVSKLFAEFDS-TEIPNMIGLRRRQLFIHDGIQIHLYDFAEKSD   59
               : * : ::.      *: :*   :    *     .   ***** ...: :*: :.   ..
```

Figure S4. Comparison of the amino acid sequences of TcmI, AbxD, StmH, and StmK proteins. (a) Alignment of the complete amino acid sequence of TcmI, AbxD, and the N-terminus and C-terminus of StmH and StmK. (b) Alignment of the N-terminus and C-terminus of StmH and StmK with each other. Among the three amino acids that play a role in controlling and catalyzing cyclizaion and aromatization in TcmI and AbxD, R40 and H51 are conserved in both the N-terminus and C-terminus of StmH/StmK, whereas Y49 is only conserved in the N-terminus and C-terminus of StmH, and, the C-terminus of StmK of D27 is substituted with Asp.

(a)

```
StmF   MTTAPVAPPPAR----SIDQRHITLDADGIMLSARLAQPVHVPPRATIVALHGAGMSAGYFDGPAHPETSLLAVAAELGFTAVAIDRPGYGRSAGRLPYGQGVVAQSHTLAAALRRLVAQ
SetF   MTTAPVAPFTGRSADPRIDRRHLTLDADGVTLSARLARPAHVPPRATVVALHGAGMSAGYFDGPAHPETSLLTLAAELGFTAVAVDRPGYGDSAGRLPHGQGVAAQADTLAAALRGLLAR
SemF   MTTAPPAPPPPAP---VVDRRLITLDADGIPLSARLALPTHVPPRATVVALHGAGMSAAYFDGPAHPETSLLTLAAELGFAAVAVDRPGYGASAGQLPYGQGVGDQAATLSAALRSLSAF
AlpS   -------------------------------MASRTKARAARPARVTLNCLAHAGAGVASYR--------DWASAVGDGVDVHALPLPGR-EARRREPRLTTRADLLADLLPGLLTTARR
                                      :::*    .   *.*.*: .*  ** ... :           : *.   *. . *:  **   :  : *           * ..*

StmF   YETGAGIFLMGHSFGSKPALQIAADGTVPEIIGLDLSGCGAEYLVPPTAPGTRAGSWKLN--WGPLRLYPPGTFQSSIGVVAPAPERELADAAQWPTVFAALAERIRVPVRFTFAEHEAW
SetF   HETGAGIFLMGHSFGCKPALRIAADGTVPGLLGLDLSGCGAEYLAAPGAPATRAGSWKLN--WGPLRLYPPGTFQSSIDVVAPAPVRELADAAHWPTVFPALAERVRVPVRFTFAEHEAW
SemF   CAPDAGIFLVGHSFGSKPALRMAADGRVPGLLGLDVSGCGDEYLVPPTTPGTRAGSWKLN--WGPLRLYPPGTFQAGLAVVAPPPERELADAARWPEEFAALAGRVRVPVRFTFAEHEAW
AlpS   GPYALYGHSMGALVGYTLTRALADAGAPP--LFLAVGACPPPHTTTALVDGADLPDEDLLPLLDTIGSLPPGASASPGGLWRRTFLPVLRDDLRLAKSLRNAALDPVTGGPVDVPVLVFA
           . :*  .* . :  :*  *  *  : * :..*  : ... . .:  . .*   ..:  ***: :   :   .    * *  : .  :   *    .   . ..

StmF   WRRD-HQALTRLRSRLVAAPRVLIDHQPGAGHNISLGWAARAYHLRALGFAEECLHRRSPGTEQRA-
SetF   WRRD-HEALTRLRNRLAAAPRVLIDHQPDAGHNISLGWAARAYHLRALGFAEECLRRYAADGKVVHP
SemF   WRRD-APTLARLRNRLDAAPRVRIDHQPDAGHNISLGWAARSYHLRALSFAEECLRRRTGGEGRQP-
AlpS   GSDDPLAAPAALRHWQQWTTDLIELHTVSGGH----FFASSAGLAQHVGRACRDHVNAQPAGGRR--
          *  : : **    :. :   *  ..**    :*: :   : :. * .   .
```

(b)

```
SpmF   MTGPQPGGVALAPQSPSTAGAPTPTRPPSAARRPAGPAPRPAPPVPLPPWTPSPGVRQVTLAAGDGITLSGLLALPRGERPRATVLALHGA GTSAAYFDGQAHPGTSLLTLAADLGYAVL
JuiF   -------------------------------------------- MTVAQPAVAGGVRGISL-DADGITLSALLALPPQAPCRAVIVALHGA GMSSAYFHGSAHPDTSFLGLATSLGFGVV
HlmB   ----------------------MTKAPP------KAPPTAPPTAPPTAPAAVSAKVGRITL-QAGGITVSALLARPLYAPPRATVVALHGA GMSAAYFHGQTRPETSLLTLAADLGFAAV
LinF   ----------------------MTAA------------------GSAVAARPPATVRRITL-DADGTPLSALLALPEHTPPRATVVALHGA GMSAAYFHGTARPDTSFLALAADLGFAAV
SemF   ----------------------MTTAPP--------------APPP---PAPVVDRRLITL-DADGIPLSARLALPTHVPPRATVVALHGA GMSAAYFDGPAHPETSLLTLAAELGFAAV
StmF   ----------------------MTTAPV--------------APPPAR----SIDQRHITL-DADGIMLSARLAQPVHVPPRATIVALHGA GMSAGYFDGPAHPETSLLAVAAELGFTAV
```

```
SetF  ----------------------MTTAPV--------------APFTGRSADPRIDRRHLTL-DADGVTLSARLARPAHVPPRATVVALHGAGMSAGYFDGPAHPETSLLTLAAELGFTAV
                                                                          ::*  ..*  :*.  ** *     **.::****** *:. **.* ::* **:* :*:.**: .:
SpmF  ALDRPGYGLSAARLPQGLPVAEQAATVRAALRGHAALHDTGAGVLVLAHSFGGKVALRMAADDRGDPSDPVLLGLDVSGCGERYARPLAELTGPAGGVRWSGHWGPLRLYPPHTFRASAR
JuiF  AVDRPGYGRSARQLPEGQGVADQATTLGAALRFLAARYDTGSGLFLLAHSFGGKAALRIAADR----AVPELLGLDVSGCGAEYAVPLGDRE-PHGRQSRRLNWGPLRLYPPGTFRASGG
HlmB  AVDRPGYGLSSAELPRGQTAAEQAATLTAALGDLADRYDTGAGLFLLAHSFGGKPALRMAAEG----TLPALLGVDVSGCGDAYTAPPTEPG-T-RDGGWRRSWGPLRLYPPGTFRSGAA
LinF  ALDRPGYGLSARRLPEGQGVMEQATTIAAAVRDLTARHLPGAGVFLLAHSFGGKPALGIAADG----LVPGLLGLAVSGCGDEYLVPPTATT-G-RAAQWRLNWGPLRLYPPGTFRASGA
SemF  AVDRPGYGASAGQLPYGQGVGDQAATLSAALRSLSAFCAPDAGIFLVGHSFGSKPALRMAADG----RVPGLLGLDVSGCGDEYLVPPTTPG-T-RAGSWKLNWGPLRLYPPGTFQAGLA
StmF  AIDRPGYGRSAGRLPYGQGVVAQSHTLAAALRRLVAQYETGAGIFLMGHSFGSKPALQIAADG----TVPEIIGLDLSGCGAEYLVPPTAPG-T-RAGSWKLNWGPLRLYPPGTFQSSIG
SetF  AVDRPGYGDSAGRLPHGQGVAAQADTLAAALRGLLARHETGAGIFLMGHSFGCKPALRIAADG----TVPGLLGLDLSGCGAEYLAAPGAPA-T-RAGSWKLNWGPLRLYPPGTFQSSID
      *:****** *: .** *  .  *: *: **:          .:*::::.**** * ** :* *:        * ::*: :****  *                 ********* **::.
SpmF  MVSPVPPRELRDAARWPGAFAALAGRVRVPVRFTFAEYEALWHCDAPALARLRAGLRAAPRVSVDRQPGAGHNISLGWAARAYHLRALAFADDCLRR---------------
JuiF  VVAPVPSLELADAARWPGDFAGFAGRVRVPVRFTFAEHEFWWQHDARAVARLRSRLSGAPRVVTDHQPDAGHNISLGWSARAYHLRVLGFVEECLRWRESRQRATPEEQEGT
HlmB  VVARVPPGELADAARWPAEFGRLAGRIRVPVRFTFAEYETWWRHDPPALDRLRSRLSAAPRVVIEHQPDAGHNISLGWAARAYHLKALGFLEESMRHHGVGRESRT------
LinF  VVAPAPPRELADAGRWPETFRRIGGRIRVPVRFTFAEHEHWWRHDRRAVAQLRSRLHAAPRVLVDHQPDAGHNISLGWTARAYHLGVLGFVADCLRPDGDGKEHPA------
SemF  VVAPPPERELADAARWPEEFAALAGRVRVPVRFTFAEHEAWWRRDAPTLARLRNRLDAAPRVRIDHQPDAGHNISLGWAARSYHLRALSFAEECLRRRTGGEGRQ-P-----
StmF  VVAPAPERELADAAQWPTVFAALAERIRVPVRFTFAEHEAWWRRDHQALTRLRSRLVAAPRVLIDHQPGAGHNISLGWAARAYHLRALGFAEECLHRRSPGTEQRA------
SetF  VVAPAPVRELADAAHWPTVFPALAERVRVPVRFTFAEHEAWWRRDHEALTRLRNRLAAAPRVLIDHQPDAGHNISLGWAARAYHLRALGFAEECLRRYAADGKVVHP-----
      :*:  *  ** **.:**  *  :. *:**********:*  *: *  :: :**  * .****  ::**.*********:**:*** .*.*  :.::
```

Figure S5. Sequence alignment of AlpS and biaryl polyketide type II TEs. AlpS and setomimycin type II TEs (a) and their respective biaryl polyketide TEs (b). The conserved GHSMG motifs are marked by azure colors, and the Ser-His-Asp triad is highlighted in red. Sequences included AlpS (AAR30163.1), SemF (MCW8383170.1), StmF (QTI46206.1), SetF (EPH44821.1), LinF (ANS69114.1), SpmF (MCI3903327.1), JuiF (QNL10618.1), and HlmB (AXL88811.1).

(a)

```
AspB -------------MTTTAT-------LTYPFH-DWSQELSPRYAQLRASDAPV-CPVVSEGTGDHLWLATRYAAAVELLEDPRLSSEAAIASGAPRQEPVELRAPGTRADGV----AMLRE   95
6KZS -------MENTSVQNKET------VRNCPFDYAHELEFDPQLRQLLTEEPVSRIRMA--YGEGEAWLVTRYEDVRTVTTDRRFSRSAVLGRDFPRMTP-----EPIVQAESINLMDPPAS  106
StmI -------MANADIDETNELVSFPIQRTCPFA------IPPVYTKFREESPIS---QVVLPDGGKAWLVTKYDDVRAVMANPKLSSDR-RAPDFPVVVPGQNA-ALAKHAPFMIILDGAEH  102
OxyA GIDPFTMFEEINVVRAAEL----H-RRDRFD------PVPQLRSLMAEGPLTTLGTEESPGGRTAWLATGYDEIRQVLSSDD-FSAR-LL------YGG--TAAGITWPGFLTQYDPPEH  99
OxyB ------MSE----DDPRPL---HI-RRQGLD------P---ADELLAAGALTRVTIGSGADAETHWMATAHAVVRQVMGDHQQFSTR-RRWDPRDEIGGKGIFRPRELVGNLMDYDPPEH   96
                                    :                .:                   *:.* :       :  .

AspB AGLRSVLADGLGPRAVRRHQGWINDLAETLMSALASREGTFDLAADFVEPLSSALVSRTLLGELSADERDLLAHCADTGLRFCGVTHEEQVHAFTQMHEFFLEHARRLAGTPGEHLLKLI  215
6KZS SRLRGLVAKSFTPRRVEQMRGGTQRVVDRLLDEMEEEGSPADFVARVSAPLPLITICE-ALDIPEADRPWLRAHA-MTMMNVGAAGKQDAVRAKAELRGYFQELTADRRRSPGEDLISTL  224
StmI AAARRPVISEFSVRRVAAMKPRIQEIVDGYIDEMLKLPKPVDLNQVFSLPVPSLVVSE-ILGMPYEGHEYFMELA-EILLRRTTDE-QGRIAVSVELRKYMDKLVEEKINNPGDDLLSRQ  219
OxyA TRLRRMVAPAFAVRRMQKFQPQVERVVQDSLDAIEALGGPVDFVPRFGWSVATTATCD-FLGIPRDDQADLARSL-HASR-TERSG-KRRTAAGNKFMTYMNKMTARTRRDPGDDMFGVV  215
OxyB TRLRRKLTPGFTLRKMQRMAPYIEQIVNDRLDEMERAGSPADLIAFVADKVPGAVLCE-LVGVPRDDRDMFMKLC-HGHLDASLSQ-KRRAALGDKFSRYLLAMIARERKEPGEGMIGAV  213
 :  *  :   : * :      : :.:  :. :      *:   .   :    .   :.    ..  :              :       ::  ::          **: ::

AspB AEAPVDHGPLSDEALAEAGSLLVVAGFPTSSGFLCGALLTLLRHPDAVQELHAHPERVPSAVEELLRYTPLS-TGSVKRMATEDLEIDGVRIKVGEVVMVSLEAVNHDPDAFEDPDVFRP  334
6KZS ATARDGDELLDDDELAVMAMVLLITGQDTTTYQLGNIAYTLLTRPDLLRSLRAEPQRLPRTLEELLRHIPFRKGVGIPRIALEDVELSGVLIKAGDVVHVSYLTANRDSAKFDRPDELDP  344
StmI IELQRQEGGIDRPALASLCLLVLLAGHETTANMINLGVFSMLTKPELLAEIKADPSKTPKAVDELLRFYTIP-DFGAHRLALDDVEIGGFLIRKGEAVIASTFAANRDPAVFDDPEELDF  338
OxyA VRE--YGDEITDAELTGVAAFVMGAGADQVARFLAAGAWLMADDPEQFALLREKPDTVPDWLDEVIRYLTTD-EKTHPRVATDDVRIGDHLIKAGDTVTCSLLAANRRNFPR-PEDRFDI  331
OxyB VAE--YGDDATDEELRGFCVQVMLAGDDNISGMIGLGVLAMLRHPEQIDAFRGDEQSAQRAVDELIRYLTVP-YSPTPRIAREDLTLAGQEIKKGDSVICSLPAANRDPALAPDVDRLDV  330
                        A/GGxxT motif                           ExxR motif

AspB GREGPMHFGFGRGRHFCPGNRLARCVIEATVRAVA-RRPGLRLAVAPEEISWHEGLFFRRPRAIPATW  401
6KZS DRPTIPHMTFGWGAHHCLGAPLATMELEVAFSTLLTRFPALRLDVPPEDVSWNTTSIWRYPLALPVTW  412
StmI GRDARHHVAFGYGPHQCLGQNLGRLELQVVFDTLFRRLPDLRLAVPEEELSFKSDALVYGLYELPVT-  405
OxyA TRVRPEHLAFGHGIHHCLGRSLAELVFRTAIPALAHRFPTLRLAEPHR--EIRLGPPPFDVEALLLDW  397
OxyB TREPIPHVAFGHGVHHCLGAALARLELRTVFTELWRRFPALRLADPAQDTEFRLTTPAYGLTELMVAW  398
```

FGHGXHXCLG/GXXXC motif

(b)

```
JuiI  MSE---ITAESVPEFPILRTCPFSVPETYRELEGTGGRIHRVRMSDGRPAWLVTKHDDARAVLSDARFSSEKLRPGFPELSPGGLKALTYFSPFLVNMDGPEHAQARRAVLGEFSVRRIN  117
SemI  MTNTDIDETSELVSFPIQRTCPFAIPPVYTKFRE-EAPVSQVVLPDGGKAWLVTKYDDVRAVMSNPKLSSDRRKKDFPVVVPGQNAALAKHAPFMIILDDPEHAEARRPVISEFSVRRVA  119
SetI  ------------------------MPPVYTKFRE-ESPITQVVLPDGGKAWLVTKYDDVRAVMANPKLSSDRRAPDFPVVVPGQNAALAKHAPFMIILDGAEHAAARRPVISEFSVRRVA  95
StmI  MANADIDETNELVSFPIQRTCPFAIPPVYTKFRE-ESPISQVVLPDGGKAWLVTKYDDVRAVMANPKLSSDRRAPDFPVVVPGQNAALAKHAPFMIILDGAEHAAARRPVISEFSVRRVA  119
SpmI  MSDAPVQETAGPIRFPITRTCPFAIPETYERLRE-EEPVSRVEMSDGRHAWMLTRHADVRAALVDPRLGSDRSDPGYPSISSGGKSAFAHFAPFMISLDGPAHSAARSPVISEFSMRRVN  119
LinI  MLNLDPEEVAKLPHYPVTRRCPYEIPEVYTRLRT-EEPVSKVVMSDGQPVWFLSRYEDVRAVLSDPRFGADRLAEGFPNLALGQREGLSKQPKFMISMDGAEHSAVRRRVISDFSVRRVA  119
                                :* .* .:.      : :* : **  .*::::: *.* *.: : ::.:::   .:* :  *   .::    *:: :*.  *: .*  *:.:**:**:

JuiI  AMRPRIRRIVDAAIDRMLEQPGRPVDLAAHLSQPVPTLVLSAFLGVPAEDLDAIERNTGKMLHEARTEDEQRAAAEALHAHLDLVIAAKEENPGDDFLSRQIDRSRREHGDAADRFELAS  237
SemI  AMKPRIQEIVDHFIDEMLKLP-RPVDLNQVFSLPVPSLVVSEILGMPYEGHEYFMELAEVLLRRTTDEQGRINISVQLRAYMDKLVEEKIANPGDDLLSRQIELQRR-NGG-IDRPQLAS  236
SetI  AMKPRIQEIVDGFIDDMLKMP-KPVDLNQVFSLPVPSLVVSEILGMPYEGHEYFMELAEILLRRTTDEQGRIAVSVELRKYMDKLVEEKIENPGDDLLSRQIELQRQ-QGG-IDRPQLAS  212
StmI  AMKPRIQEIVDGYIDEMLKLP-KPVDLNQVFSLPVPSLVVSEILGMPYEGHEYFMELAEILLRRTTDEQGRIAVSVELRKYMDKLVEEKINNPGDDLLSRQIELQRQ-EGG-IDRPALAS  236
SpmI  TLRPRIQEIVDEAIDKILELP-RPVDLVQHLALIVPRLVITERIGASPDYLERFYALAAGMLQRSTTGEERDAIAREMRANMDRLVAEKEADPGDDLLSREIARQRAETGD-VDRPGLAS  237
LinI  ELRPTIQKLVDDCVDRILDLP-QPVDLVAELALPVPTLLLAELVGANHADHAYFIDLVHRMLWRKTSGEERVQISIGLRKYFDDLIAEKEAHPGDDLISRQIALQREETGE-IDREGLNS  237
       ::* *:.:**  :* :*.  * :****   ::  ** *:::  :*         :   .  :* .     : :   :  ::  :* ::  *  .****::**:*  .*   *   **  * *

JuiI  LVQLLQIAGHASSAAMISLSVLTLLSEPEQLKQLTADPSRTPAAAEELLRFLSITDTGPLRLALEDVEIGGVRIRAGDGVLIPTLPANRDAGAFPDPDRFDIGRLPGTRHVAFGYGAHQC  357
SemI  LCLLVLLAGHETTANMINLGVFSMLTRPELLDVIKADPSKTPKAVDELLRFYTIPDFGAHRLAMDDVEIGGVLIRKGEAVIASTFAANRDPAVFDDPEELDFSRD-SRHHVAFGYGPHQC  355
SetI  LCLLVLLAGHETTANMINLGVFSMLTKPELLAEIKADPSKTPKAVDELLRFYTIPDFGAHRLALDDVEIGGVLIRKGEAVIASTFAANRDPAVFDDPEELDFGRD-ARHHVAFGYGPHQC  331
StmI  LCLLVLLAGHETTANMINLGVFSMLTKPELLAEIKADPSKTPKAVDELLRFYTIPDFGAHRLALDDVEIGGFLIRKGEAVIASTFAANRDPAVFDDPEELDFGRD-ARHHVAFGYGPHQC  355
SpmI  LAQLLLLAGHESTSEMISLGIATLLTHPEQLARMVADPSRTPAVIEELLRYFSVVEIGMGRVATEDVELGGVRIKAGEGVIASNVAANHDPRAFPDPDVFDPDRD-ARQHVAQGYGPHQC  356
LinI  LAQLLLIAGYESSASMIALGVHTFLTRPDWLAAIRTDPARTSVAVEELLRFYSILDVAAGRVALEDVEIGGETIRAGDGVMASVFAANRDPSAFPDPDRLDLERG-ARHHVAFGYGPHQC  356
```

A/GGxxT motif　　ExxR motif　　FGHGXHXCLG/GXXXC motif

```
JuiI  LGQNLVRAELQVVLDRLFHRIPDLRLATAPEALPYKYFGQFFGPVELPVTW  408
```

```
SemI  LGQNLGRVELQVVFDTLFRRLPDLRLAVPAEELNFKSDALVYGLYELPVTW  406
SetI  LGQNLGRLELQVVFDTLFRRLPELRLAVPEEELSFKSDALVYGLYELPVTW  382
StmI  LGQNLGRLELQVVFDTLFRRLPDLRLAVPEEELSFKSDALVYGLYELPVTW  406
SpmI  LGRNLARVELQTVFDTLFRRIPGLRLAVGVEELPFKYDALVHGLRELPVTW  407
LinI  LGQNLSRLELQIVFDTLFARVPTLRLAVDEADLPFKYDALAFGLYELPVTW  407
      **:** * *** *:* ** *:* ****.    * :* .  .*   ******
```

Figure S6. Sequence alignment of P450 eznymes. Sequence alignment of actinomycetes P450s and StmI (a) and their respective biaryl polyketide P450s (b). Typical P450-fold, with helix L containing the signature sequence ^{348}FGHGXHXCLG^{357} containing the proximal axial thiolate ligand of the heme iron, ^{355}Cys, is conserved, and the highly conserved ^{244}A/GGxxT^{248} motif and the ^{283}ExxR^{286} motif in the K-helix, and the ^{351}GxxxC^{355} motif in the heme binding ring is also conserved in StmI and its corresponging enzymes. All of them are marked in red. Amino acids conserved in biaryl polyketide P450 that are specific to the motif region are coloured green. Sequences included OxyA (GIF17054.1),OxyB (Q8RN04.1),AspB (WP_030881046.1), 6KZS (WP_003970936.1), JuiI (QNL10608.1), SemI (MCW8383179.1), SetI (EPH44812.1), StmI (QTI46197.1), SpmI (MCI3903336.1), and LinI (ANS69122.1).

```
FdxE    ------MKVRLDPSRCVGHAQCYAVDPDLFPIDDSGNSILAEHEVRPEDMQLTRDGVAACPEMALILEEDDAD   67
LinM    -MSGAAVRITGDPEVCTGAGQCVLTDPAMFAQDDRGVVELRTHRAEGARVPRAREAVFLCPSGALSIVED---   69
SemM    ------MRLEKDHDRCIGAGQCVRTDPRIFDQDDDGLVKVLIDRPRGTDEAAARKAVFICPAKALSLAQD---   64
StmM    ------MKLEKNHDRCIGAGQCVLTEPRIFDQDDRGLVSVLVERPQGADVAAARKAVYICPAKALSLVEE---   64
SetM    MVKEMAMKLEKDHDRCIGAGQCVLTEPRLFDQDDWGLVDILVERPRGADEAAARKAVYICPAKALSLVEE---   70
              :::  : . * * .**  .:* :*  ** *   :  .. .      :*..*  **  ** : ::
```

Figure S7. Sequence alignment of FdxE and biaryl polyketide ferredoxins. The genes which encode [3Fe–4S] or [4Fe–4S] single cluster containing ferredoxins have unusual iron–sulfur cluster binding motif sequences, CXX?XXC(X)nCP, where '?' indicates a variable amino acid residue. Rather than a cysteine residue, which is highly conserved in [4Fe–4S] clusters, alanine or glycine residues at this position in [3Fe–4S] ferredoxin. The conserved CXXA/GXXC(X)nCP motif of [3Fe-4S] ferredoxin is highlighted in red. In FdxE, the Ala residue of the CXXA/GXXC(X)nCP motif is substituted by a His residue and is coloured green. Sequences included StmM (QTI46198.1), SetM (EPH44813.1), SemM (MCW8383178.1), LinM (ANS69121.1), and FdxE (O53937.1).
